# A novel tick-borne phlebovirus, closely related to severe fever with thrombocytopenia syndrome virus and Heartland virus, is a potential pathogen

**DOI:** 10.1038/s41426-018-0093-2

**Published:** 2018-05-25

**Authors:** Shu Shen, Xiaomei Duan, Bo Wang, Liying Zhu, Yanfang Zhang, Jingyuan Zhang, Jun Wang, Tao Luo, Chun Kou, Dan Liu, Chuanwei Lv, Lei Zhang, Chenchen Chang, Zhengyuan Su, Shuang Tang, Jie Qiao, Abulimiti Moming, Cheng Wang, Abulikemu Abudurexiti, Hualin Wang, Zhihong Hu, Yujiang Zhang, Surong Sun, Fei Deng

**Affiliations:** 1 State Key Laboratory of Virology, Wuhan Institute of Virology, Chinese Academy of Sciences, Wuhan, 430071 China; 2 Xinjiang Key Laboratory of Biological Resources and Genetic Engineering, College of Life Science and Technology, Xinjiang University, Urumqi, 830046 China; 3 Center for Disease Control and Prevention of Xinjiang Uygur Autonomous Region, Urumqi, 830001 China; 4 School of Medicine, Wuhan University of Science and Technology, Wuhan, 430065 China; 5 Hubei Collaborative Innovation Center for Green Transformation of Bio-resources, Hubei Key Laboratory of Industrial Biotechnology, College of Life Science, Hubei University, Wuhan, 430061 China

## Abstract

Tick-borne viral diseases have attracted much attention in recent years because of their increasing incidence and threat to human health. Severe fever with thrombocytopenia syndrome phlebovirus (SFTSV) and Heartland virus (HRTV) were recently identified as tick-borne phleboviruses (TBPVs) in Asia and the United States, respectively, and are associated with severe human diseases with similar clinical manifestations. In this study, we report the first identification and isolation of a novel TBPV named Guertu virus (GTV) from *Dermacentor nuttalli* ticks in Xinjiang Province, China, where TBPVs had not been previously discovered. Genome sequence and phylogenetic analyses showed that GTV is closely related to SFTSV and HRTV and was classified as a member of the genus *Phlebovirus*, family *Phenuiviridae*, order *Bunyavirales*. In vitro and in vivo investigations of the properties of GTV demonstrated that it was able to infect animal and human cell lines and can suppress type I interferon signaling, similar to SFTSV, that GTV nucleoprotein (NP) can rescue SFTSV replication by replacing SFTSV NP, and that GTV infection can cause pathological lesions in mice. Moreover, a serological survey identified antibodies against GTV from serum samples of individuals living in Guertu County, three of which contained neutralizing antibodies, suggesting that GTV can infect humans. Our findings suggested that this virus is a potential pathogen that poses a threat to animals and humans. Further studies and surveillance of GTV are recommended to be carried out in Xinjiang Province as well as in other locations.

## Introduction

Emerging pathogenic tick-borne viruses (TBVs) that can infect animals and humans have attracted much attention because of the increasing incidence of tick-borne viral diseases (TBVDs) and their significant impact on human health. In recent years, two novel tick-borne phleboviruses (TBPVs), severe fever with thrombocytopenia syndrome virus (SFTSV), and Heartland virus (HRTV), were shown to be associated with severe human diseases and resulted in fatal cases in Eastern Asian countries and in the United States^1–7^. SFTSV was first isolated from patients in China who had severe fever with thrombocytopenia syndrome disease (SFTS) with clinical signs that included fever, thrombocytopenia, and leukocytopenia, among others^4^. From 2010 to 2016, over 10,000 SFTS cases, with an average mortality rate of 5.3%, were reported in 23 provinces in China according to the Chinese Disease Prevention and Control Information System^8^. The virus was then identified in Japan and South Korea^1,2,9,10^. SFTSV infections resulted in 46 reported deaths out of 170 confirmed SFTS cases in Japan (a mortality rate of 27.1%) until 2016^2^ and 54 deaths out of 172 SFTS cases in South Korea (a mortality rate of 31.4%) until 2015^11^. HRTV was identified from patients with clinical symptoms similar to those observed for SFTS in the United States in 2009, which has a close genetic relationship to SFTSV^5^. To date, over 30 HRTV cases, including three deaths, have been reported in United States^6,7,12^. The discovery of SFTSV and HRTV suggested that TBPVs could cause severe emerging human diseases and pose a significant, potentially previously unrecognized threat to public health. Currently, increasing efforts to identify, survey, and investigate emerging TBVs will be very important to gain a better understanding of these viruses and to deal with the new or re-emerging TBVDs.

Due to the rapid development of metagenomics, increasing numbers of novel arboviruses have been or are going to be identified from various vector hosts^13^. Another novel TBPV called Hunter island group virus (HIGV) was isolated from ticks collected from an island in Australia^14^. Although HIGV infections have not been observed in local residents, its close genetic relatedness to both SFTSV and HRTV indicated the zoonotic potential of HIGV and that it should be further investigated^14^. Another TBPV that was isolated from Lone Star ticks (*Amblyomma americanum*), named Lone Star virus (LSV), was recently genetically characterized and determined to be a novel member of the Bhanja group in the genus *Phlebovirus*^15,16^. The Lone Star tick is also a major tick vector from which HRTV was isolated^17^, which is considered to be a vector that transmits many disease-causing pathogens of humans and animals^18^. Therefore, a broad investigation of viral pathogens in ticks is also important to provide insights into the diversity and evolution of TBPVs.

*Dermacentor nuttalli* is a dominant tick species in the northern Xinjiang Province, China^19^ and is an important vector of tick-borne diseases, including tularemia, rickettsiosis, anaplasmosis, brucellosis, Q fever, and babesiosis^20,21^. Livestock can become heavily infested by *D. nuttalli*, causing severe economic loss of livestock breeding and cultivation. *D. nuttalli* ticks are also aggressive to humans. In Xinjiang Province, *D. nuttalli* ticks are vectors of spotted fever group (SFG) rickettsia and infest local, individuals, causing SFG rickettsiosis^22^. However, the viral pathogens that *D. nuttalli* ticks may carry are unknown. In this study, we described a novel TBPV named Guertu virus (GTV) that was isolated from *D. nuttalli* ticks collected from Xinjiang Province, China. Phylogenetic analysis showed GTV is an intermediate species with very close evolutionary relationships to SFTSV and HRTV. We characterized the infection properties and pathogenicity of GTV through in vitro and in vivo experiments and investigated the seroprevalence of antibodies against GTV in humans. Our findings suggested GTV is a pathogen posing a potential health threat to humans.

## Results

### Isolation and identification of a novel phlebovirus from ticks in Xinjiang Province

Ticks collected from Guertu County were identified as *D. nuttalli* according to their morphology and molecular taxonomy using a partial sequence of the mitochondrial 16S rRNA gene (Figure S1). In total, 398,653 preprocessed reads were obtained from 8 pools of *D. nuttalli* tick samples by 454 sequencing. Overall, 431 reads were determined to be related to SFTSV by BLASTx comparisons and were assembled into seven contigs (three of the L segment, two of the M segment, and two of the S segment). The known SFTSV genomic sequences deposited in GenBank exhibit a nucleotide similarity of more than 90%. Interestingly, the SFTSV-related contigs generated by 454 sequencing showed only 77–86% amino acid (aa) identity to the known SFTSV sequences, suggesting that they belong to a novel phlebovirus that is closely related to but distinct from SFTSV. RT-PCR using primers specific to the S segment (C9F1 and C9R1, supplementary data) was performed to confirm that the two sample pools tested positive for the viral contig sequences (data not shown). Thus, the virus was named Guertu virus (GTV) after the location from which the ticks were collected.

**Fig. 1:**
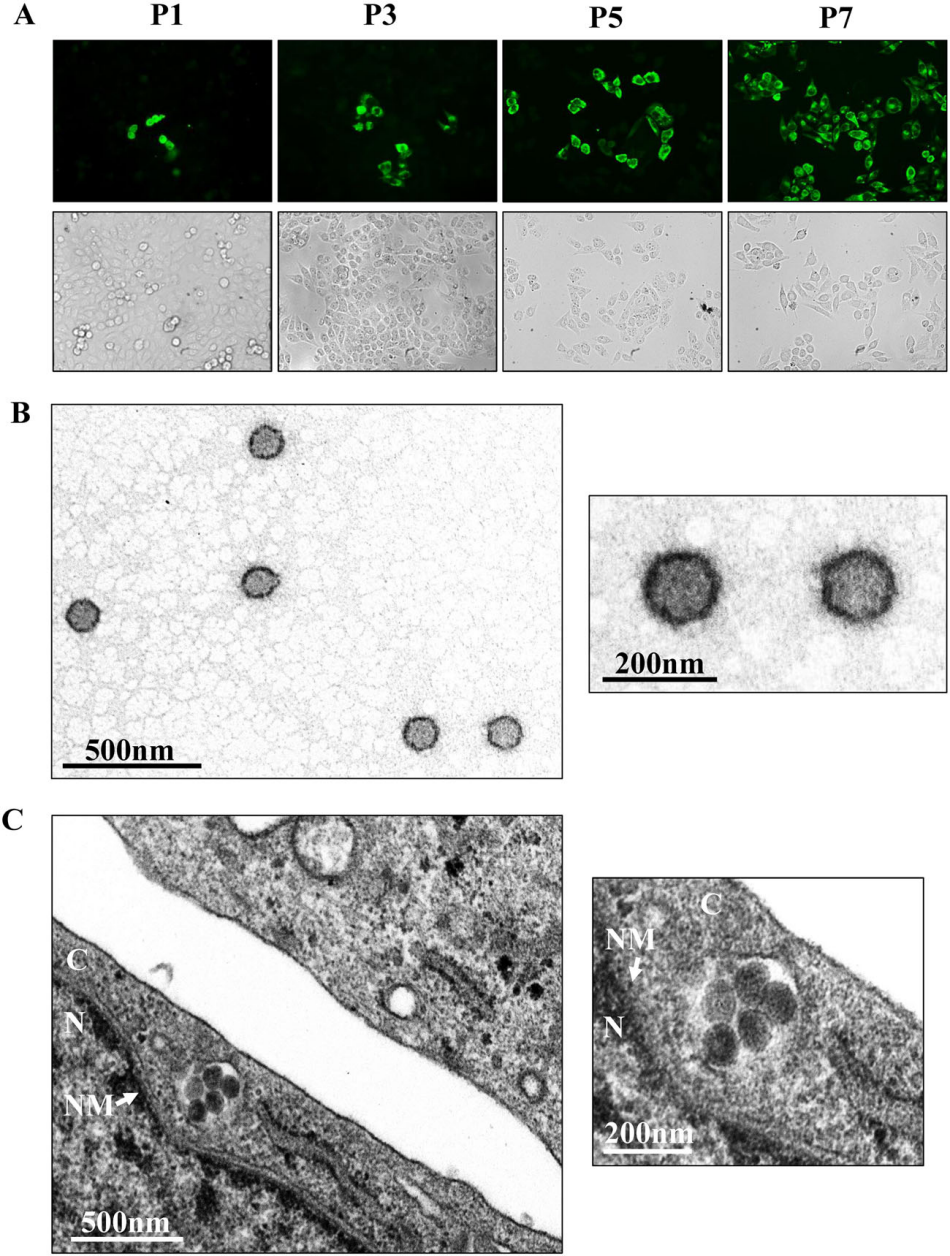
GTV isolation from ticks and EM analysis of viral particles. **a** Immunofluorescence assays to survey SFTSV infection in Vero cells from each passage. The images taken from different passages showing the virus proliferation derived from the 1:40 dilution are presented. Cells immunostained by green fluorescence indicated that they were infected by GTV. P1, the first passage; P3, the third passage; P5, the fifth passage; and P7, the seventh passage. **b** EM analysis of GTV particles purified from the cell culture supernatants. The supernatants were harvested from Vero cells infected with GTV after 5 days p.i. and were centrifuged and filtered to remove the cell debris. The clarified supernatants were subjected to ultracentrifugation. The fractions containing viral particles were harvested and used in negative-staining EM analyses. **c** The image obtained by TEM show that virus particles were located in cytoplasm of infected Vero cells. The enlarged images of interest show virus particles from (**b**) and (**c**) are shown on the right. N, nucleus; C, cytoplasm; and NM, nuclear membrane

Subsequently, GTV was isolated from the homogenates of one GTV-positive sample pool. Because the contig sequence (614 nt) for the S segment shared 86% amino acid identity to SFTSV nucleoprotein (NP) (positions 4–200 aa), we attempted to survey virus infection in each passage by immunofluorescence assay (IFA) using α-SNP to detect GTV NP expression in cells. Fortunately, we observed green fluorescence in a few cells after the first passage from both dilutions (Fig. 1a, P1). The number of infected cells increased gradually in the subsequent passages (Fig. 1a). After the 7th passage, almost all cells were infected, suggesting that GTV efficiently proliferates in Vero cells (Fig. 1a, P7). Viral RNA was detected in supernatants from different passages by RT-PCR (Figure S2). The results showed that GTV was isolated and that a higher efficiency was observed from the 1:40 dilution. Furthermore, viral particles were purified from the supernatants and visualized by negative-staining electron microscopy (EM) analysis, which showed spherical and enveloped virions with a diameter of ~80–120 nm (Fig. 1b). Virus particles were observed to cluster in the cytoplasm of infected cells (Fig. 1c).

### Molecular characterization and phylogenetic analysis showed GTV is a distinct member of the genus *Phlebovirus*

The complete genomic sequence of the GTV isolate was obtained. The genome contains three segments (large (L), middle (M), and small (S) segments), which have been deposited in GenBank under the accession numbers KT328593, KT328592, and KT328591 (strain DXM), respectively. The L segment is 6366 nucleotides in length and contains one open reading frame (ORF) encoding an RNA-dependent RNA polymerase (RdRp, 2084 aa). The M segment is 3371 nucleotides in length and contains one ORF encoding a glycoprotein precursor (G, 1063 aa), which would be cleaved into mature Gn and Gc. The S segment is 1744 nucleotides in length and contains two ORFs encoding nonstructural protein (NSs, 285 aa) and NP (245 aa) using an ambisense coding strategy, the same as SFTSV.

**Fig. 2:**
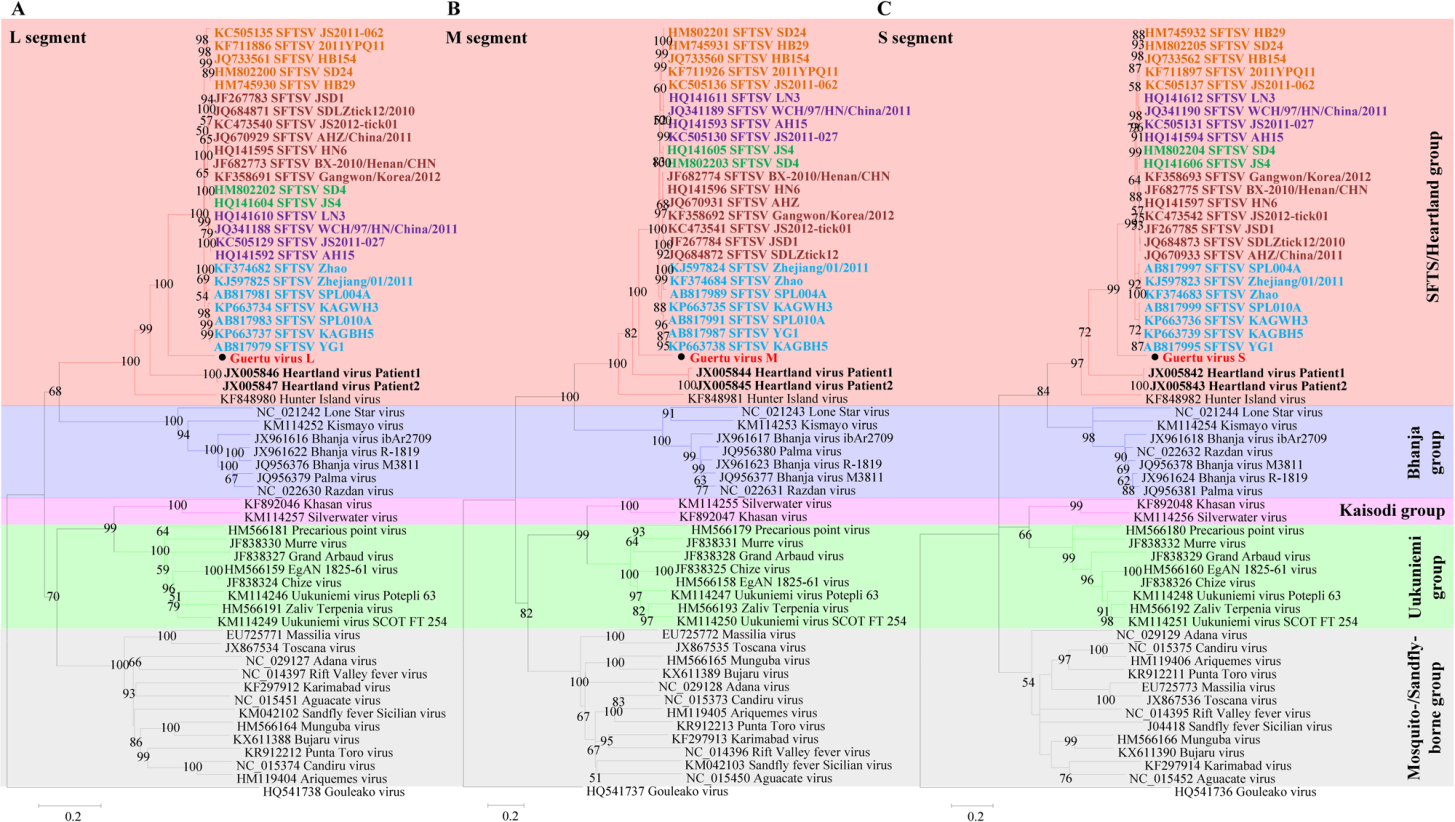
**The ML phylogenetic tree of phleboviruses built based on the complete sequences of the L (a), M (b), and S (c) segments.** The branches of five groups of Phleboviruses are labeled with different colors. GTV is labeled with a black solid circle and shown in bold red characters. HRTV sequences are indicated by bold black characters. SFTSV sequences are shown in bold characters, and five genotypes are distinguished by different colors: C1 in green, C2 in chocolate, C3 in khaki, C4 in purple, and J in cyan. Trees were constructed using Mega 5.0 and tested by the bootstrap method with 1000 replications. Bootstrap values greater than 50% are shown at each node

From these results, it is evident that GTV is a novel member of the genus *Phlebovirus* in the family *Phenuiviridae* and that it is most closely related to SFTSV and HRTV (Fig. 2 and Table S1). Phleboviruses can be divided into five groups according to their phylogenetic relationships^23^. Among the five groups, SFTSV, GTV, HRTV, and HIGV comprise the SFTS/Heartland group. To better show that GTV is distantly related to SFTSV, SFTSV strains from five genotypes (C1, C2, C3, C4, and J)^24^ were included in the maximum-likelihood (ML) trees. It was noted from the trees that HIGV diverged first, followed by HRTV, GTV, and SFTSV, making GTV an intermediate species between SFTSV and HRTV. Comparisons of nucleotide and aa sequences showed that GTV was more similar to SFTSV than HRTV (Table S2). The terminal sequences (11–17 nt) in the UTRs from the L, M, and S segments were conserved among GTV, SFTSV, and HRTV (Figure S3), although the identities of the UTR sequences varied from 37 to 86%. The internal untranslated regions (IUTRs), flanked by NSs and NP ORFs from S segments, differed in length, and the GTV IUTR shared 51–52% identity to those of SFTSV and HRTV (Figure S3 C). The viral proteins RdRp and NP were more conserved than G and NSs among the viruses (Table S2 and Figure S4). Alignments of viral proteins allowed for conserved putative functional domains to be identified and revealed that GTV is more closely related to SFTSV than HRTV. First, GTV, SFTSV, and HRTV RdRp all have specific Bunyavirus RdRp domains, including an endonuclease, DUF3770 (unknown function), and a core Bunyavirus polymerase domain (BUNV_Polymerase domain), as well as the motifs important for RNA binding and nucleotide addition (Figure S4A). Second, the crystal structures of SFTSV Gn and Gc, which have been recently characterized, revealed important structural characteristics, residues, and motifs^25,26^. Sequence alignment showed that five N-glycosylation sites, cysteines responsible for disulfide bonds formation and multimerization, and two hydrophobic loops important for fusion are fully conserved among the GTV, SFTSV, and HRTV glycoproteins (Figure S4B). Only two residues (F286 and C287, SFTSV numbering) in the neutralizing antibody epitope (α6) of SFTSV are fully conserved. The key residue (K288 in SFTSV) responsible for hydrogen bonds and salt bridges with an antibody is conserved in GTV Gn (K285) (Figure S4B), suggesting that GTV Gn is likely to be recognized by the antibody derived from SFTSV. Third, eight sites responsible for intermolecular interactions and five sites for RNA binding^27^ are fully conserved among GTV, SFTSV, and HRTV NPs, while two sites for each function are conserved in GTV and SFTSV but varied in HRTV (Figure S4C). This result indicated that the GTV NP may function more similar to SFTSV NP. A PXXP motif that was suggested to play roles in NSs-induced inclusion body (IB) formation and function in SFTSV NSs (P66-X-X-P69)^28^ are conserved in GTV and HRTV NSs (Figure S4D), indicating the potential of IB formation with similar functions.

### GTV can infect and replicate in animal-derived and human-derived cells

**Fig. 3:**
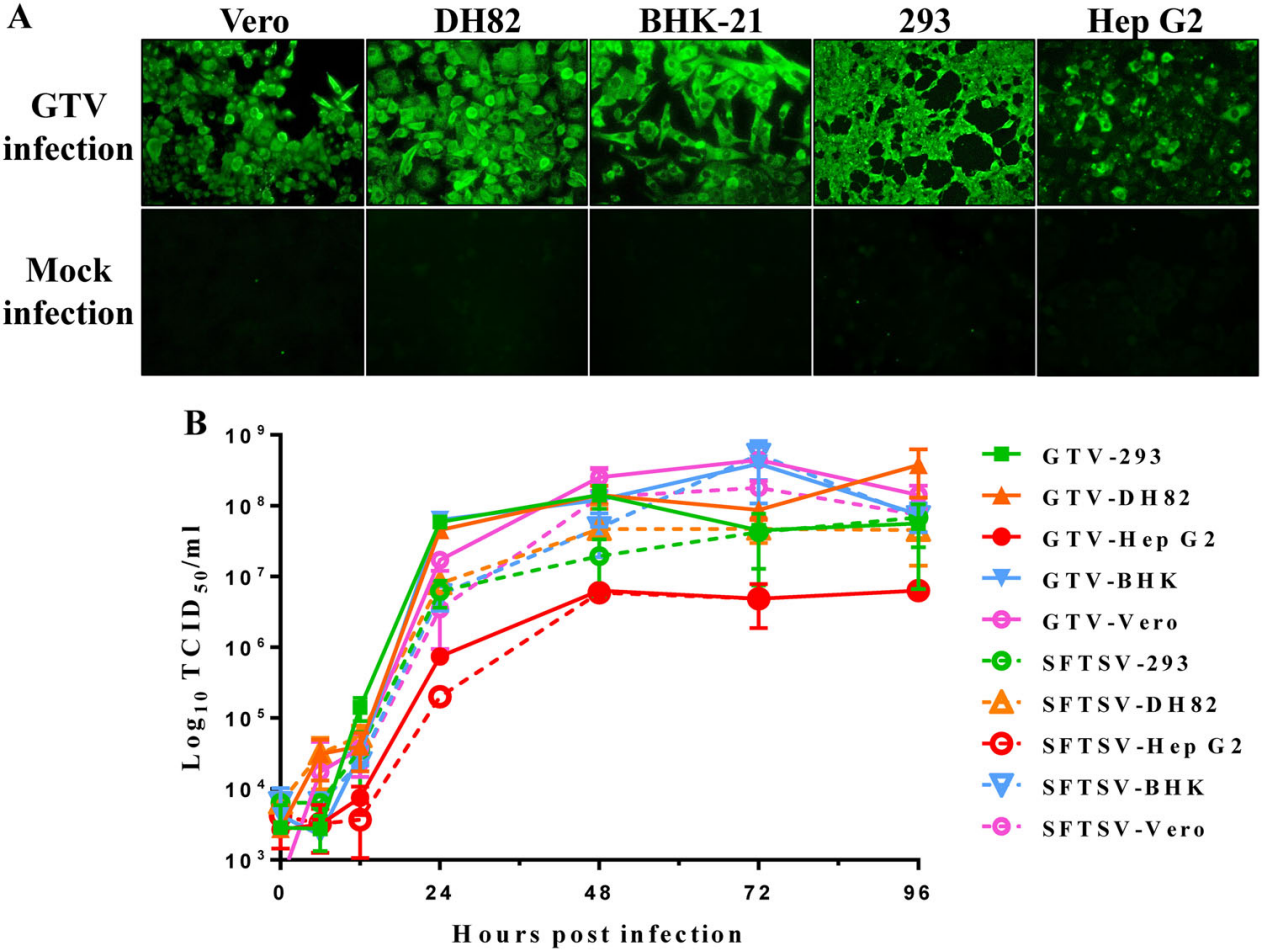
The susceptibility of different cell lines derived from different hosts to GTV. **a** Immunofluorescence assay to detect the susceptibility of different cell lines derived from monkey (Vero), dog (DH82), mouse (BHK-21), and different human tissues (293 and Hep G2 cells). The mock infection was used as the negative control. **b** The one-step growth curve of GTV and SFTSV in different cell lines. Cells were infected with GTV or SFTSV at an MOI of five TCID_50_ units per cell. Supernatants were harvested at the indicated time points, and the viral titers in supernatants were determined as described

As GTV is genetically related to SFTSV and HRTV, both of which can infect animals and humans, we investigated the ability of GTV to infect and replicate in cell lines derived from different hosts. As shown by IFAs, green fluorescence (indicating GTV NP expression) was observed in Vero (Monkey), DH82 (Dog), BHK-21 (Mice), 293 (Human), and Hep G2 (Human) cells, suggesting that all tested cells could be infected by GTV (Fig. 3a). A one-step growth curve of GTV in these cells showed that progeny viruses in supernatants were produced at high titers from Vero, DH82, BHK-21, and 293 cells at 48 h post infection (h p.i.), and the rate of virus production was maintained during the following 48 h (Fig. 3b). Although Hep G2 cells could be infected by GTV, significantly less progeny viruses were produced from this cell line (*P* < 0.05) (Fig. 3b), probably due to a less efficient replication in Hep G2 cells. SFTSV growth properties were characterized in parallel (Fig. 3b). Similar to GTV, SFTSV could infect the five tested cell lines and yielded comparatively higher infectious progeny viruses from Vero, DH82, BHK-21, and 293 cells. Lower SFTSV titers were also detected from Hep G2 cells, similar to that observed for GTV, and generated a similar growth curve to GTV.

### GTV NP can rescue SFTSV NP function for virus replication

**Fig. 4:**
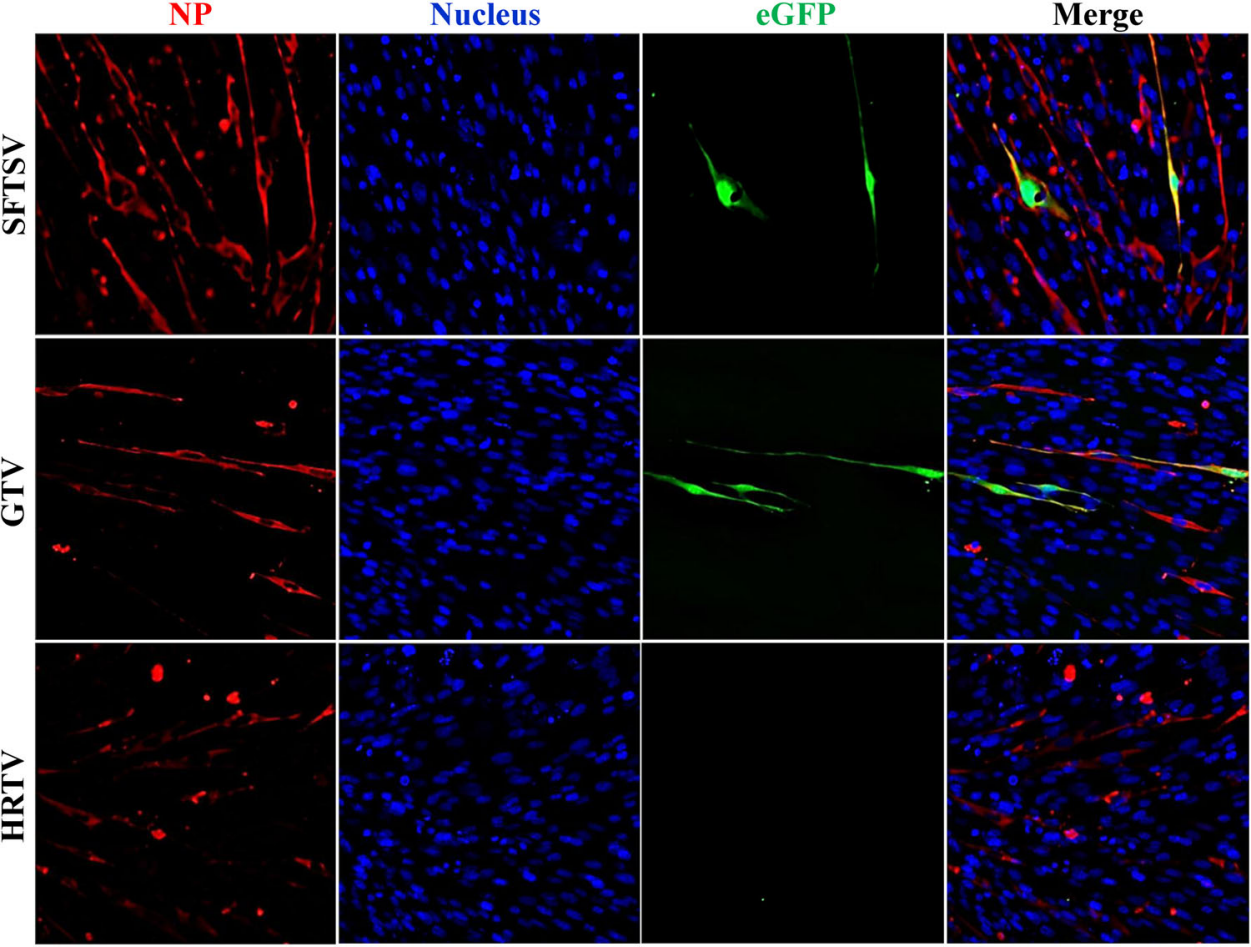
Reporter assays using the SFTSV replicon system with the substitution of SFTSV, GTV, or HRTV NP, respectively. The reporter assays were performed using the *egfp* reporter plasmid system. NP expression in each test was immunostained with α-SNP or an HA tag. Cell nuclei were stained with Hoechst 33258. The eGFP expression was visualized under an inverted fluorescence microscope

GTV NP had high aa similarity (88%) to SFTSV NP (Figure S4C and Table S2), indicating it may be a functional homolog of SFTSV NP. We used an SFTSV replicon system to test the function of the GTV, SFTSV, and HRTV NPs in viral replication. SFTSV, GTV, and HRTV NPs were expressed in cells in each test (Fig. 4). As expected, eGFP expression was visualized in cells expressing SFTSV NP, while eGFP expression was also observed in GTV NP-expressing cells. However, eGFP expression was not induced in cells expressing HRTV NP (Fig. 4). The results showed that GTV NP could rescue SFTSV RNA replication, while HRTV NP could not. The ability of the GTV NP to rescue the SFTSV NP function was further confirmed using the SFTSV replicon system with a *luciferase* gene reporter plasmid, which resulted in a comparable level of luciferase activity triggered by GTV and SFTSV NPs (Figure S5).

### GTV NSs can form IBs that differ in morphology from SFTSV NSs-induced IB structures

**Fig. 5:**
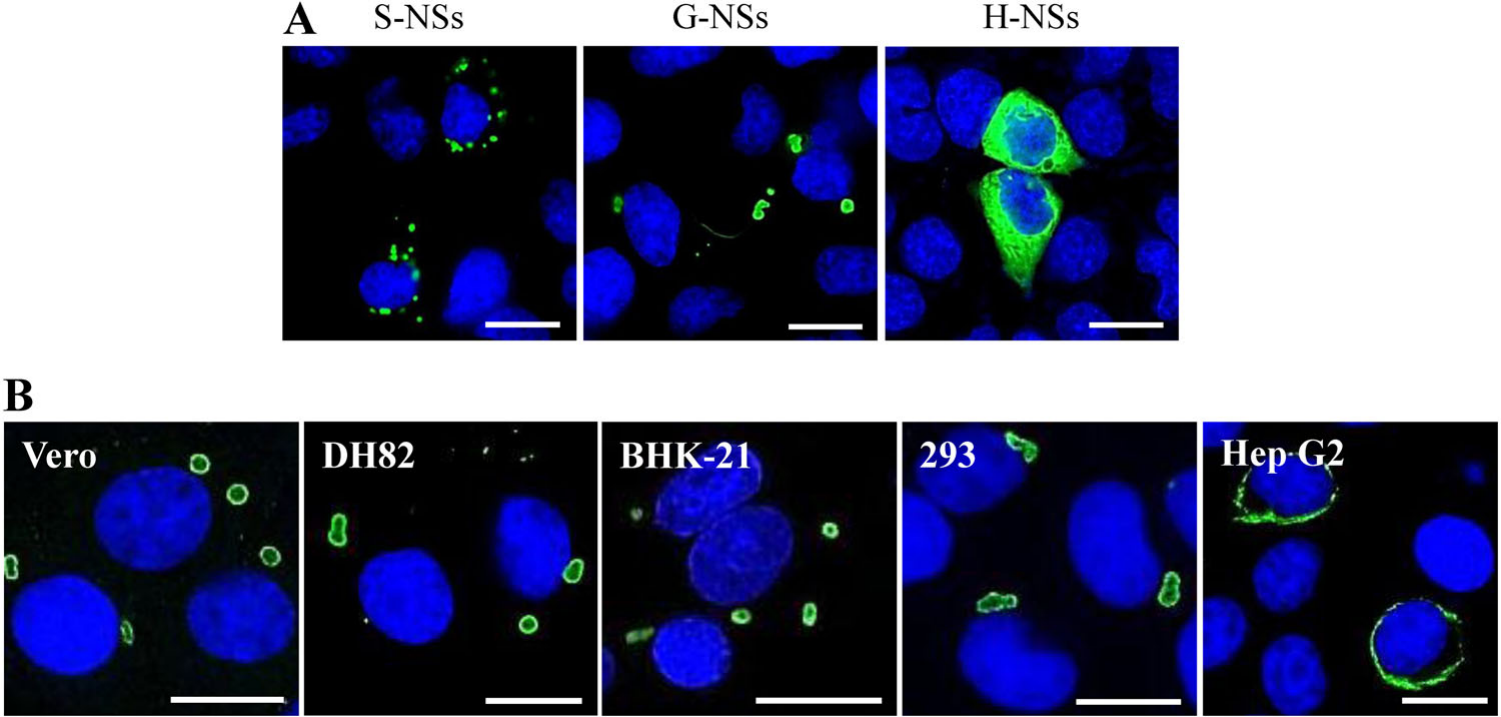
Immunofluorescence assay to characterize the NSs-induced IB formation in cells. **a** The NSs distribution in cells was observed by IFA in 293 cells transfected with NSs expression plasmids. G-NSs, GTV NSs; S-NSs, SFTSV NSs; and H-NSs, HRTV NSs. **b** GTV NSs-induced IB formation was observed in cell lines from different hosts. NSs expression in cells was detected by IFA. Bars, 15 μm

To the best of our knowledge, SFTSV is a unique phlebovirus in that NSs functions in virus replication and assembly and in the viral suppression of innate immunity by forming compact IB structures^28–31^. Consistent with previous findings^28,31,32^, SFTSV NSs formed IBs of heterogeneous sizes that were scattered in the cytoplasm of cells, as revealed through transfection assay, whereas HRTV NSs could not form IBs and were dispersed in the cytoplasm of transfected cells (Fig. 5a). Interestingly, fewer IBs of larger sizes and heterogeneous shapes formed by GTV NSs were observed in the cytoplasm of a single 293 cell (Fig. 5a). Therefore, the conserved PXXP motif present in NSs among the three viruses, which was suggested to be important for IB formation for SFTSV NSs, may not be the crucial factor in determining IB formation for phleboviruses.

IB formation was characterized in different cell lines with GTV infection (Fig. 5b). Similar IB structures were observed in GTV-infected Vero, DH82, BHK-21, and 293 cells. A few IB granules were observed in the cytoplasm of a single cell, presenting an incompact structure with GTV NSs that were primarily located on the surface of IBs. Typical IB structures were not observed in Hep G2 cells. Instead, a fiber-like structure was formed by GTV NSs around the nucleus in these cells.

### GTV infection suppressed type I IFN signaling

**Fig. 6:**
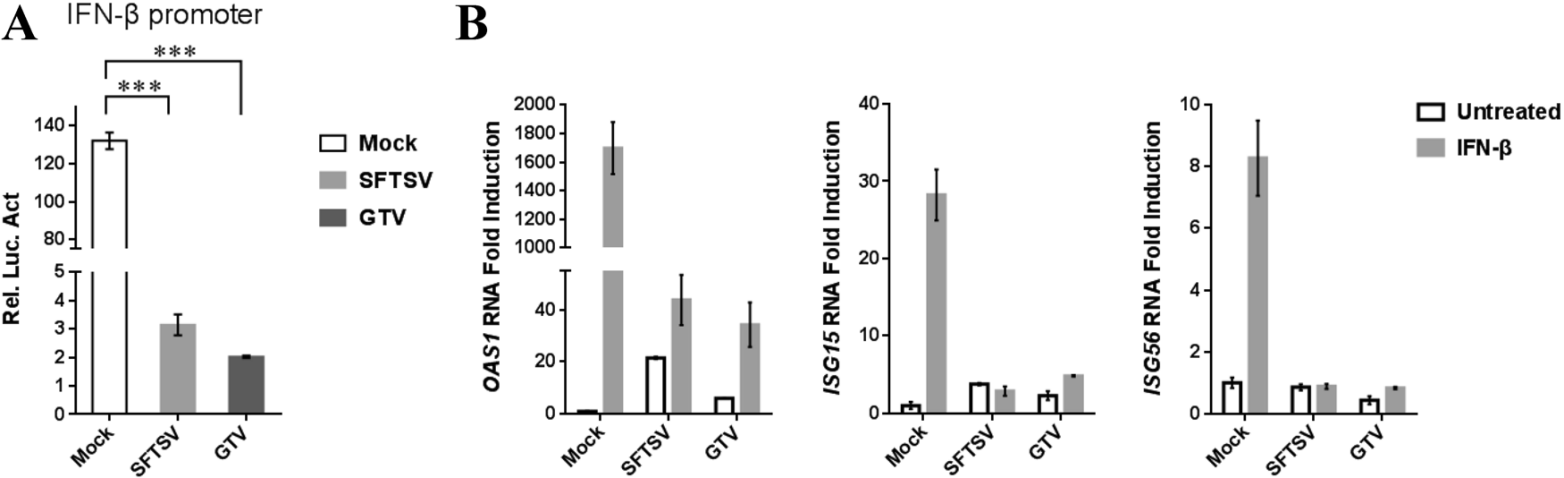
GTV infection suppresses type I IFN signaling. **a** GTV infection inhibits IFN-β-triggered activation of the ISRE promoter. 293T cells were co-transfected with the IFN-β reporter and pRL-TK plasmids. At 12 h post transfection (p.t.), cells were mock infected or infected with SFTSV or GTV. After 8 h, cells were treated with SeV for another 16 h, after which luciferase activities were measured. The relative luciferase activities (Rel.Lucif.Act.) were expressed as the firefly luciferase activity normalized to Renilla luciferase activity. **b** GTV infection suppressed IFN-induced gene expression. 293T cells were mock infected or infected with SFTSV or GTV for 24 h and then treated with IFN-β (200 U/ml) or left untreated for 10 h. Expression of ISGs was measured by qRT-PCR. Each test was performed in triplicate. Data are presented as the means ± SD

The effect of GTV infection on type I IFN signaling suppression was investigated by a DLR gene assay. The ISRE promoter could be activated by IFN-β treatment, which resulted in a high level of luciferase activity in the mock-infected cells (Fig. 6a). The luciferase activity was significantly reduced in the SFTSV-infected cells due to SFTSV NSs ability to suppress IFN signaling^29^. Similarly, the luciferase activity also remarkably reduced in GTV-infected cells (Fig. 6a). These results suggested that GTV infection can suppress type I IFN signaling, similar to SFTSV. The expression of several IFN-β-induced ISGs, *oligoadenylate synthetase 1* (*OAS1*), *ISG15*, and *ISG56*, were further examined by qRT-PCR analyses. As expected, GTV infection greatly suppressed the expression of induced ISGs, as efficiently as SFTSV (Fig. 6b). The results confirmed that the IFN signaling was inhibited by GTV infection.

### GTV can cause pathological lesions in mice

**Fig. 7:**
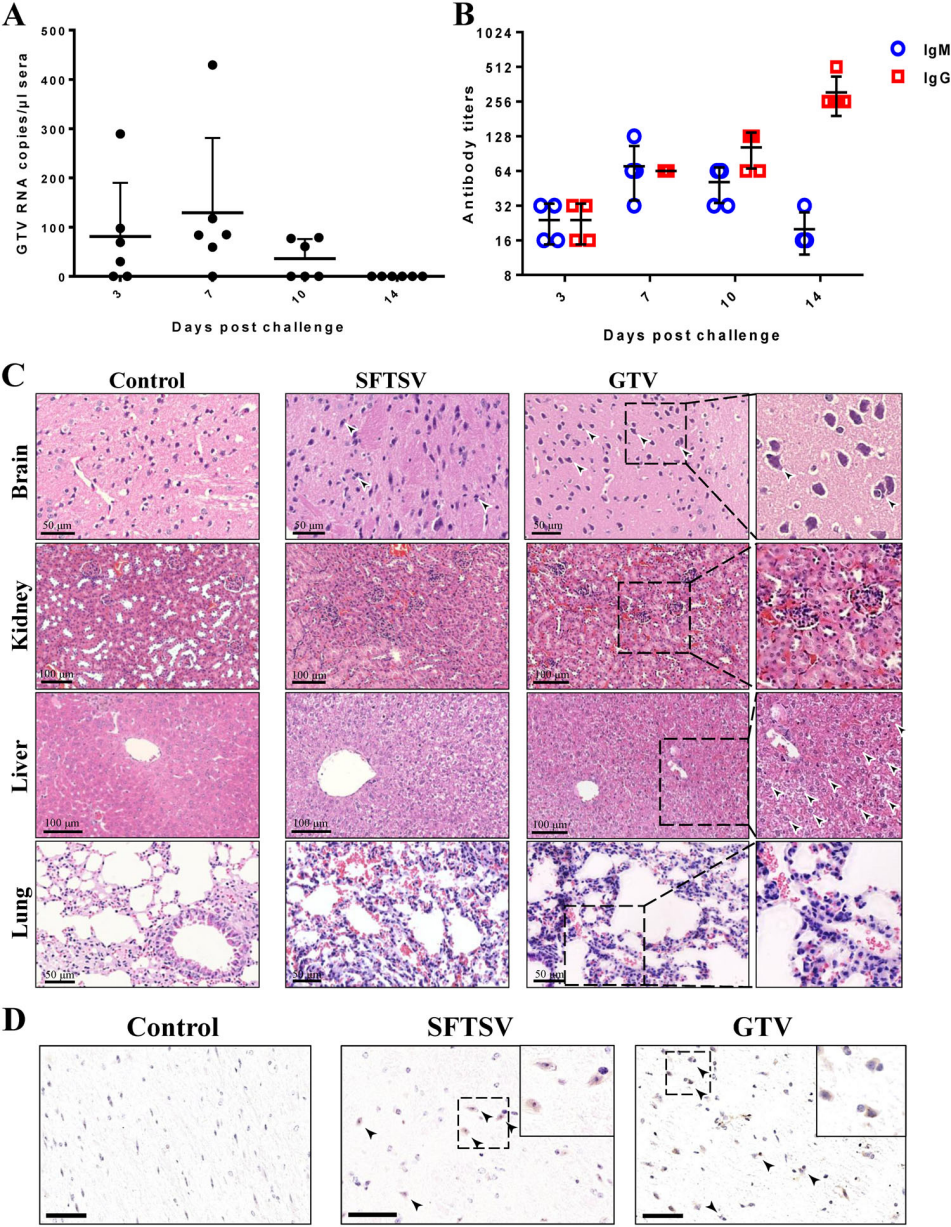
The pathogenicity of GTV infection in C57/BL6 adult mice. **a** qRT-PCR detection of GTV RNA copies in sera collected on the indicated day after challenge. Each dot represents the RNA copies per microliter of sera from each mouse. Data are presented as the means + SD. **b** IgM and IgG antibody expression in sera from mice collected on the indicated day after challenge. Sera were twofold serially diluted using an end-point dilution assay. The titers are expressed as reciprocals of the dilution, the OD values from which are three time higher than the negative control. The antibody titers are shown with the corresponding symbols with means ± SD. **c** The pathological changes in organs from mice infected with GTV or SFTSV. Representative H&E staining tissue sections are presented, including for the brain, kidney, liver, and lung. Areas of interest are enlarged on the right, and significant changes are indicated by arrows. **d** Identification of GTV and SFTSV antigen expression in mouse brains. GTV NP and SFTSV NP expression was identified by IHC in the brains of mice at day 14 post infection. Representative images are shown, and the areas of interest are enlarged on the right. The neurons expressing viral antigen or being sieged by microglial cells are indicated by arrows. Bars, 50 μm

The pathogenicity of GTV infection to animals was investigated using C57BL/6 mice that were challenged intraperitoneally (IP). Significant clinical signs, including changes in body temperature and weight losses were not detected during the 14 days after challenge, and all the mice survived until they were killed. GTV RNA was detected in sera collected on days 3, 7, and 10 by qRT-PCR, suggesting that viremia was induced in mice as a result of GTV infection (Fig. 7a). On day 14, viremia had been cleared from all mice (Fig. 7a). A humoral immune response was induced in all the tested mice (Fig. 7b). IgM expression was detected on day 3, increased on day 7, after which it gradually decreased after 10 days. Low levels of IgG were expressed on day 3 and increased remarkably on day 14. Neutralizing antibodies against GTV were detected in the serum samples collected on day 14 (Table S3), which might be important for virus clearance in sera.

The pathological changes induced by GTV infection in organs were evaluated by H&E staining (Fig. 7c). The mouse brains exhibited neuronophagia, which is a typical sign of viral encephalitis. The mouse kidneys exhibited renal tubulointerstitial hemorrhaging, edemas of renal tubular epithelial cells, and mesangial thickening. The lesions in livers primarily presented ballooning degeneration of hepatocytes. The pathological changes in lungs included hemorrhaging in alveolar spaces and inflammatory cell infiltration. Furthermore, IHC analyses showed viral antigen expression in the neurons of mice brains on day 14, some of which were associated with smaller cells, likely to be glial cells (Fig. 7d, arrows). IHC staining for GTV antigen expression was negative in the kidneys, livers, lungs, intestines, and spleens of mice (data not shown). The results suggested that GTV can be delivered to the nervous system and that the brain was the target organ for GTV infection in mice. Similar pathological changes in the brains, kidneys, livers, and lungs of mice were also observed in C57BL/6 mice infected with a high dose of SFTSV (Fig. 7c). SFTSV antigen expression in neurons was also observed in IHC experiments (Fig. 7d), but this expression was not detected in mice challenged by lower viral dose (10^5^ TCID_50_ or 10^5^ PFU per mouse) and was only observed in SFTSV-infected IFAAR^−/−^ mice^33–35^. Cross-neutralization tests showed that the neutralizing antibodies in serum samples from GTV-infected mice could prevent SFTSV infection in Vero cells, and the serum samples collected from SFTSV-challenged mice also had neutralizing antibodies against both SFTSV and GTV (Table S3).

### Investigation of the prevalence of GTV in ticks and a serological survey in humans revealed the potential risks of GTV infection to humans

The prevalence of GTV in groups of *D. nuttalli* ticks was investigated by nested RT-PCR. In addition to the 2 GTV-positive pools used for Roche 454 sequencing, 4 other pools tested positive for GTV RNA from the remaining 68 pools. PCR products from the four pools were sequenced, showing that partial S segment sequences from three pools (hsf-2, hsf-3, and cggl-14, 295 bp in length) were identical to the isolated virus (strain DXM), while one fragment (J-67) shared very high similarity (99.3%) to strain DXM. A phylogenetic tree built with the sequences from the PCR products showed that GTV sequences clustered together, further confirming that GTV is distinct from SFTSV and HRTV (Figure S6) and indicating that a population of GTV strains may be circulating among *D. nuttalli* ticks in Guertu County.

To investigate the potential risks of GTV infection to humans, 465 serum samples were collected from individuals with an average age of 42.43 in Guertu County and examined by IFA (Table 1 and Figure S7). Among these individuals, 186 are herdsmen with frequent contact with cattle, sheep, and dogs, and 278 are farmers that primarily work in fields and croplands. Males and females accounted for 48.6% and 51.4% of individuals sampled, respectively. The IFA results showed that green fluorescence could be visualized from 92 serum samples, with a positive rate of 19.8% among the total samples (Figure S7), 50 of which came from herdsmen (positive rate of 26.7%) and 42 were from farmers (positive rate of 15.1%) (Table 1). The highest positive rate (7.5%) was observed for individuals with ages of 31–45. Neutralizing antibodies against GTV were identified in three serum samples, two from herdsmen (H3 and H5) and one from farmers (F3). The end-point titers of the neutralizing activity were 2^5^ for H3 and H5 and 2^6^ for F3 (Table 1). Hubei Province in central China had not been reported as a habitat of *D. nuttalli* ticks and is therefore not supposed to be an epidemic area of GTV. The 15 serum samples from this region all tested negative to GTV as assessed by IFA (Figure S7).

**Table 1:** Background information and results of serological examination of the human serum samples from Guertu County

Occupations	Serum samples	Gender		Ages and percentage		Positive antibodies against GTV detected by IFA (%)	Neutralization tests	
		Male (%)	Female (%)				GTV	End-point titer
Herdsmen	186	95(50.8%)	92(49.2%)	5–80	186	55(26.9%)		
				<18	7(3.8%)	3(1.6%)	0	
				18–30	34(18.3%)	9(4.8%)	0	
				31–45	74(39.8%)	20(10.8%)	2	2^5^ for both
				46–60	56(30.1%)	6(3.2%)	0	
				>60	15(8.1%)	6(3.2%)	0	
Farmers	278	131(47.1%)	147(52.9%)	15–83	278	42(15.1%)		
				<18	8(2.9%)	1(0.4%)	0	
				18–30	41(14.7%)	7(2.5%)	0	
				31–45	101(36.6%)	15(5.4%)	1	2^6^
				46–60	103(37.1%)	18(6.5%)	0	
				>60	25(9.0%)	1(0.4%)	0	
Total	465	226(48.6%)	239(51.4%)	5–83	465	92(19.8%)	3	
				Average	42.43			
				<18	15(3.2%)	4(0.9%)	0	
				18–30	75(16.1%)	16(3.4%)	0	
				31–45	175(37.6%)	35(7.5%)	3	
				46–60	159(34.2%)	24(5.2%)	0	
				>60	40(8.6%)	7(1.5%)	0	

## Discussion

This study is the first to report on the isolation and identification of a novel TBPV, named Guertu virus (GTV), from *D. nuttalli* ticks and genetically related to two pathogenic TBPVs, SFTSV and HRTV, which can infect animals, including cattle, sheep, dogs, cats, pigs, chickens, and hedgehogs^11,36,37^ and cause symptomatic or even lethal illness in humans^4,5^. Sharing a recent common ancestor, GTV evolved to become an intermediate species between HRTV and SFTSV, making it very important to carry out further investigations on GTV traits and infection in cells and potential pathogenesis.

We characterized the general functions and traits of GTV NP and NSs, which showed similar functions to SFTSV. GTV and SFTSV NPs shared high amino acid similarity with all conserved residues important for their function. Second, GTV NP rescued SFTSV replication by replacing SFTSV NP, as revealed by reporter assays. Therefore, GTV NP is a functional analog of SFTSV NP, and GTV and SFTSV might have a similar mechanism of RNA replication. SFTSV used to be a unique member of phleboviruses in that it is known to be able to form IBs induced by NSs and can suppress innate immunity^28^. In this study, we determined that GTV NSs could also form IBs but presented shapes and sizes different from those induced by SFTSV NSs. No noticeable IB formation could be induced by HRTV NSs^32^. These results suggested that GTV is closely related to SFTSV with respect to viral protein function and suppression of innate immunity^29^. SFTSV infections have been detected in many domestic animals^11,36,37^, and it can infect and replicate in various human and mammalian cells with different efficiencies^38^. Serological investigations have suggested that HRTV (or HRTV-related) infections occur in wild and farmed animals^39,40^, whereas only DH82 and Vero E6 cells were used for HRTV isolation and culture^5^. In this study, similar growth properties of SFTSV and GTV were observed in the tested cell lines derived from monkey (Vero), dog (DH82), mice (BHK-21), and human (293 and Hep G2). Hep G2 cells were less efficient than the other tested but could still sustain both GTV and SFTSV replication (Fig. 3). As the GTV IBs presented an atypical structure in Hep G2 cells, we speculated that the atypical IBs may result in the partial activity of GTV NSs, which would affect GTV growth in cells. To aid in virus infection and proliferation, SFTSV infection could circumvent host antiviral responses by suppressing type I IFN signaling^28,41,42^, and SFTSV and HRTV NSs are the primary virulence factors that function as IFN antagonists^31,32^. We also determined that GTV infection could suppress type I IFN signaling, similar to SFTSV. Whether the IBs induced by GTV NSs played roles in the molecular mechanisms of IFN antagonism, and consequently had impact on virus replication and production, should be further investigated, which will expand our knowledge of the TBPV pathogenesis related to NSs.

The pathogenicity of emerging TBPVs, such as SFTSV and HRTV, has not been fully understood. Previous findings showed that mice with SFTSV and HRTV infections did not show any clinical signs of disease^33,34,43,44^, whereas illness and lethal diseases could only be induced in mice with an interferon (IFN) receptor knockout^34,35,43^. Histopathological changes and viral antigen expression were observed in tissues, including the livers, kidneys, and spleens of IFNAR^−/−^ mice infected with SFTSV^34,35^ as well as IFN-α/β/γ receptor-deficient mice infected with HRTV^43^. Similarly, no clinical symptoms were observed in GTV-infected mice. Although viremia was induced in these animals, it was cleared in the following days. IgM and IgG expression was induced and had neutralization activity against GTV, which might be responsible for the elimination of viremia. Remarkable pathological changes in GTV-infected mice tissues were observed, including in the brain, kidney, liver, and lung, suggesting that GTV is an infectious pathogen with the potential to infect animals. The brain was shown to be the target organ of GTV infection, as viral antigen expression was observed in this organ, revealing that GTV can invade the nervous system and may be associated with encephalitis. Through the surveillance of the clinical symptoms of SFTS patients in China and Korea, neurological symptoms and encephalitis occurrence were reported from some of the SFTS patients, higher rates of which were observed in fatal cases^45–47^. This result indicates that SFTSV may be an encephalitis-related phlebovirus. However, very limited SFTSV infection was observed in the brains of IFNAR^−/−^ C57BL/6 mice^35^ and was not described in other infected mice that were challenged with a lower viral dose (10^5^ TCID_50_ or 10^5^ PFU per mouse)^33,34,44,48^. In our study, pathogenic lesions and viral antigen expression were observed in the brains of mice challenged with a high dose of SFTSV (1 × 10^7^ TCID_50_ per mouse), revealing the likelihood that SFTSV is associated with encephalitis. Previously, knowledge about HRTV pathogenesis in vitro was limited. Pathological lesions and viral antigen expression were observed in the spleens, livers, and intestines of HRTV-infected Ag129 mice (α/β/γ receptor knockout)^43^. Acute inflammation was observed in the spleens, lymph nodes, livers, and lungs of hamsters deficient in STAT2 function^12^. Nevertheless, a recent study of an HRTV-associated death without comorbidities claimed novel clinical and pathological findings, including the detection of viral antigens in the patient’s brain^6^. Encephalitis was also described in a single human case due to natural infection with another TBPV called Bhanja virus (BHAV)^49^. RVFV is another phlebovirus associated with late-developing encephalitis in mice, and in cases of human infections where RVFV antigens were detected in neurons and some small glial cells^50,51^. The exact cause of the phlebovirus-induced encephalitis requires further evaluation. More research on GTV pathogenesis will offer insights into the pathogenesis of TBPVs and reveal the potential threats from the emerging TBPVs to humans.

This study is also the first to report the isolation of a novel TBPV with pathogenic potential and health risks in Xinjiang Province, China, where no TBPVs have been previously reported^52^. Epidemiological investigation revealed that *D. nuttalli* ticks are the vector of GTV in Guertu County and identified 6 GTV-positive pools among 76. One drawback of our study is that we failed to determine the prevalence of GTV in individual ticks. Nevertheless, antibodies against GTV were detected in 19.8% of serum samples from the local residents, 3 of which had neutralization activity against GTV, suggesting the ability of GTV to infect humans. In Guertu County, herdsmen may be exposed to greater risk factors than farmers, such as GTV-infected animals, because they had a higher seroconversion rate (26.9%). Individuals aged from 31 to 45 are the major labor force that works outdoors, which may raise their risks to GTV infection and resulted in a higher seroconversion (7.5%). In addition, three serum samples were determined to have neutralization activity against GTV, which further suggested that GTV can infect humans. Thus, the potential risks from GTV infection to humans were previously unrecognized. Moreover, GTV RNA was detected in tissues from *Citellus undulatus* (one of the major hosts of the larval and nymphal *D. nuttalli* ticks^53^) collected in identical locations (unpublished data), indicating the possible transmission of GTV from ticks to animal hosts. Currently, it is unclear whether GTV can cause infectious disease in humans. Patients with HRTV infection have very similar clinical manifestations to SFTS patients, including fever, fatigue, diarrhea, and thrombocytopenia, as well as elevated levels of liver-associated transferases^4,5^. Thus, we would predict that the disease caused by GTV might present similar symptoms. In recent years, a few SFTS cases have been reported in Xinjiang Province, although these cases were not experimentally confirmed^8^. Because of the antigenic similarity of SFTSV and GTV, it is likely that positive sera against SFTSV found in serological studies might be actually reactive to GTV, which would result in the misdiagnosis of SFTSV seroprevalence. Therefore, broad epidemiological surveys of GTV are now strongly recommended in Xinjiang Province as well as other areas of China, which will reveal the distribution of GTV and provide an extensive assessment of the potential risks of GTV infection. It is also necessary to investigate GTV characteristics for a better understanding of the mechanisms of TBPV infection and replication as well as its association with host factors, which will benefit the design of strategies to respond to emerging GTV-related disease in the future.

## Material and methods

### Tick samples, viral metagenomics, and complete genome sequencing

In 2014, a total of ~13,000 unfed ticks were collected from the wild in a field in Guertu County, Wusu City, located in the Western Tianshan Mountainous areas in Xinjiang Province, China (Supplementary data). The ticks were classified as *D. nuttalli* and grouped into 76 pools of 100–200 individuals each according to the sampling locations. Eight pools of tick samples were randomly chosen and used for library preparation, which were sequenced on a Roche 454 GS FLX system (GS-FLX, Roche Applied Science) for metagenomic sequencing (Supplementary data). The complete genomic sequences of GTV were obtained by gap-filling RT-PCR with specific primers designed based on corresponding contig sequences. The terminal sequences were confirmed by rapid-amplification of cDNA ends (RACE)^54^.

### Virus isolation, purification, and electron microscopy

To isolate viruses from the homogenates of ticks, clarified supernatants from one pool testing positive for viral RNA were diluted with Dulbecco’s Modified Eagle’s Medium (DMEM, GIBCO) supplemented with 2% fetal bovine serum (FBS, GIBICO). Vero cells were incubated with two different dilutions (v/v = 1:4 and 1:40) (Supplementary data). Virus infection in cells from each passage was monitored by IFAs using α-SNP as a primary antibody. The cells from the first passage were diluted three-fold for the next passage. The subsequent passages were conducted identically, with IFA analyses performed to survey virus infections. The production of viruses released into the supernatants was evaluated using RT-PCR (Supplementary data).

The virus particles were concentrated and purified from the culture supernatants, and the fractions containing viral particles were collected and applied to grids for negative-staining EM as previously described^55^. The GTV-infected cells were fixed with 2.5% (w/v) glutaraldehyde in 0.1 M sodium phosphate to prepare ultrathin sections. The virus particles in cells were observed by transmission electron microscopy (TEM).

### Cell infection assays and one-step growth curve analysis

The cells used in this study were purchased from the American type culture collection (ATCC) (Supplementary data). Cells were infected with viruses at a multiplicity of infection (MOI) of 5 TCID_50_ units per cell at 37 °C. IFA was performed to detect virus infection in cells using α-SNP, α-SNSs, or α-SGn as primary antibodies.

To generate a one-step growth curve, 50 μl of supernatants were harvested from each infection at indicated time points. The virus titers in supernatants were determined by an end-point dilution assay using Vero cells^55^.

### Reporter assays to detect the ability of GTV NP to substitute for SFTSV NP in viral RNA replication

To examine the ability of the GTV NP to substitute for SFTSV NP in viral replication, the SFTSV replicon system was constructed using a reporter plasmid with the polymerase I system (pRF42) as previously described^56^. The reporter plasmid, NP expression plasmids, and RdRp expression plasmid were constructed as described in the supplementary materials and methods. BHK-21 cells were co-transfected with 1 μg reporter plasmid containing *egfp* or *luciferase* (pRF42-SFTSV M-egfp/Luciferase), 50 ng pRL-TK control plasmid, 500 ng SFTSV RdRp expression plasmid, and 500 ng NP expression plasmid from GTV, SFTSV, or HRTV. At 48 h p.t., luciferase activities were measured and expressed as the firefly luciferase activity normalized to the Renilla luciferase activity. The activity from the cells co-transfected with the SFTSV NP expression plasmid was set as 100%. Activities from other groups were expressed as the values normalized to the SFTSV NP group. The eGFP expression in cells was visualized under an inverted fluorescence microscope.

### Quantitative analyses of GTV inhibition on type I interferon signaling

The dual luciferase reported (DLR) gene assays were performed to detect IFN-β-triggered luciferase activities in mock- or virus-infected 293 T cells. To assess the expression of type I IFN-stimulated genes (ISGs), real-time RT-PCR was performed as previously described^29^. The actin mRNA levels were detected as an internal control for real-time RT-PCR using the primers AF: 5′-CTGAACCCCAAGGCCAACC-3′, and AR: 5′-TTCATGAGGTAGTCAGTCAGGT-3′. The relative mRNA levels were calculated using the 2^−△△CT^ method and were shown as the relative fold change by normalizing to the untreated-control samples.

### Virus infection of mice, serology, histopathology, and immunohistochemistry

Five adult C57/BL6 mice were challenged IP with 100 μl of virus-containing supernatants per mouse (containing 8 × 10^7^ TCID_50_ GTV or 1 × 10^7^ TCID_50_ SFTSV), and each mouse in the control group was inoculated IP with 100 μl DMEM containing 2% FBS. Sera were collected from each mouse on days 3, 7, 10, and 14 after challenge. All mice were killed on day 14 and tissues were collected and then fixed in 4% paraformaldehyde for 24 h, after which routine paraffin embedding was performed. Serial sections (5-μm thick) were used for hematoxylin-and-eosin (H&E) staining. Immunohistochemical (IHC) staining was performed using α-SNP as a primary antibody to detect the presence of virus antigens in mouse tissues as previously described^57^. The virus loads in sera were determined by real-time RT-PCR (Supplementary data). The titers of IgG and IgM antibodies were examined by ELISA using SFTSV mouse IgG and IgM ELISA kits (NewZongke, Wuhan, China), respectively. Cross-neutralization assays were performed to determine the neutralizing activity for both SFTSV and GTV using serum samples collected from GTV or SFTSV-challenged mice on day 14 (Supplementary data).

### Human serum samples and serological examination

Serum samples were collected from 465 local residents in Guertu County, and their background information, including their gender, age, and occupations, is listed in Table 1. GTV-infected Vero cells and uninfected cells were seeded in 96-well plates. Next, IFA was performed using the serum samples diluted (1:20) in PBS with 1% BSA to investigate the seroprevalence of antibodies against GTV. The incubation of each serum sample with healthy cells was performed using the same dilution as used for the negative control. The serum samples from GTV-challenged and SFTSV-challenged mice and the polyclonal antibodies α-SNP and α-SGn were used as positive controls. Fifteen serum samples from healthy residents (aged 18 to 30) in Hubei Province in central China were used as control sera for IFA. Serum samples without obvious signal compared to healthy cells and presenting green fluorescence, primarily in the cytoplasm of GTV-infected cells, were considered to contain antibodies against GTV. Neutralization tests were performed with antibody-positive serum samples detected by IFA as previously described^4^.

### Bio-informatics and biostatistics analyses

Sequence alignments were carried out using Mega 5.0 (MEGA) and edited by ESPript 3.0 (SBGrid)^58^. The ML phylogenetic trees were constructed and edited using Mega version 5.0 and were tested with 1000 bootstrap replicates. Predictions of glycoprotein domains and topologies were performed using the SignalP 4.1 server (http://www.cbs.dtu.dk/services/SignalP/) for signal peptide and cleavage sites, the TMHMM server version 2.0 was used to predict trans-membrane helices (http://www.cbs.dtu.dk/services/TMHMM-2.0/), and NetNGlyc 1.0 (http://www.cbs.dtu.dk/services/NetNGlyc/) was used to predict N-glycosylation sites. Putative functional domains of RdRp were predicted by comparing the sequences with other phleboviruses using the Pfam database (pfam.xfam.org/). Significant differences (*P* values) were analyzed by one-way analysis of variance (ANOVA)^59^.

### Ethical statement

All animal experiments were performed in an animal biosafety level 2 lab according to the animal welfare and the related regulations of lab activities as described in the Directory of Pathogenic Microorganisms Transmitted among Humans issued by the Chinese Ministry of Health. The procedures for animal experiments were approved by the ethics committees of Wuhan Institute of Virology, the Chinese Academy of Sciences (Approval number: WIVA33201702). The studies involving human serum samples were also reviewed and approved by the institutional ethics committees (Approval number: WIVH01201501). Written informed content was provided by adult participants and parents on behalf of child participants under 18 years old.

## Electronic supplementary material

Supplementary data

Figure S1

Figure S2

Figure S3

Figure S4

Figure S5

Figure S6

Figure S7 revision

Figure S8 revision

Figure S9 revision
