# Supplementary material for: A novel tick-borne phlebovirus, closely related to severe fever with thrombocytopenia syndrome virus and Heartland virus, is a potential pathogen
Source: Emerg Microbes Infect. 2018 May 25;7:95. doi: 10.1038/s41426-018-0093-2 (PMC5970217; doi:10.1038/s41426-018-0093-2)
Supplement: Supplementary file 2 — Figure S1 [file 41426_2018_93_MOESM2_ESM.docx]

**Supplementary data**

**Supplementary materials and methods**

**Cells and viruses**

Vero (African green monkey kidney cells, ATCC number: CCL-81), DH82 (dog macrophages, CRL-10389), BHK-21 (baby hamster kidney, CCL-10), 293 (human embryonic kidney, CRL-1573), 293T (human embryonic kidney, CRL-11268), and Hep G2 (human hepatocellular carcinoma, HB-8065) cells were purchased from ATCC and cultured in Dulbecco’s Modified Eagle’s Medium (DMEM, GIBCO) supplemented with 10% fetal bovine serum (FBS, GIBICO). For virus isolation and infection assays, the cells were maintained in DMEM containing 2% FBS.

Sendai virus (SeV) was propagated, sub-packaged, and preserved as described ^[1](#_ENREF_1)^. SFTSV (strain HBMC5) [^2^](#_ENREF_2) was expanded in Vero cells in a biosafety level 2 laboratory as described [^1^](#_ENREF_1) and was used for infection assays and animal experiments.

**Plasmids, antibodies, and reagents**

The open reading frames (ORFs) encoding GTV non-structural protein (NSs) and nucleoprotein (NP) were amplified by reverse transcription (RT)-PCR from GTV genomic RNA and were cloned into the expression vector pCAGGSP7, respectively. The partial sequence of GTV S segment (1065-1576 nt) was amplified by RT-PCR from GTV RNA genome with primers GTVSpF: 5’-CATTAGGGAAGAAGACAGAG-3’ and GTVSpR: 5’- TAAAGAGGTGGGAGGTGA-3’ using 2×Rapid Taq Master Mix (Vazyme Biotech, Nanjing, China) and cloned into pT-Easy vector, which was used as the DNA template to produce RNA standards by *in vitro* transcription assay. The sequence of HRTV NP ORF was synthesized according to the sequence in GenBank (accession number: KJ740146) [^3^](#_ENREF_3) and was cloned into pCAGGSP7. The ORF of SFTSV NP and RNA-dependent RNA polymerase (RdRp) was amplified by RT-PCR from SFTSV genomic RNA and cloned into pCAGGSP7. The reporter plasmids (pRF42-SFTSV M-egfp/luciferase) used to report SFTSV RNA replication were constructed by inserting the cassette of the *egfp* or the firefly *luciferase* ORF in the reverse direction flanked by the un-translational regions (UTRs) of SFTSV M segment into the plasmid pRF42 under the control of polymerase I promoter and terminator [^4^](#_ENREF_4). The SFTSV NSs expression plasmid (pCAGGSP7-SFTSV NSs), the firefly luciferase reportor plasmid for IFN-β promoter, and the Renilla luciferase control palsmid (pRL-TK) were described previously ^[1](#_ENREF_1)^. The HRTV NSs expression plasmid in fusion with HA tag (pCAGGSP7-HRTV NSs-HA) [^5^](#_ENREF_5) was kindly provided by Dr. Yunjia Ning. Transfection assays of the NSs expression plasmids in 293 cells were performed using Lipofectamine 3000 Reagent (Thermo Fisher) according to the manufacturer’s instructions.

To generate anti-SFTSV Gn polyclonal antibody (α-SGn), a fragment of partial sequence of SFTSV glycoprotein open reading frame (ORF) (786bp in length corresponding to Gn positions from 190 to 451 aa) was amplified by RT-PCR using the cDNA of SFTSV strain WCH-2011/HN/China/isolate97 [^6^](#_ENREF_6) as the template and the primers GbF: 5’-CGCGGATCC(*Bam*H I)ACATTTCTGGAGCTGAAAAGCTT-3’ and GbR: 5’-CGCCTCGAG(*Xho* I)CTTTGCAGGGTAGCACTGGG-3’. PCR was performed using 2×Rapid Taq Master Mix (Vazyme Biotech) according to the manufacture’s instruction ^[7](#_ENREF_7)^. The PCR products were sequenced and cloned into the plasmid pET-28a using the restriction endonuclease sites *Bam*H I and *Xho* I to generate the expression plasmid pET-28a-SFTSV GnP. The protein expression in *E*.*coli* BL21 cells was detected by SDS-PAGE. Then ptrotein was purified and inoculated into rabbit to obtain α-SGn as described [^7^](#_ENREF_7).

IFAs in this study were performed as previously described ^[2](#_ENREF_2)^. Primary antibodies used in this study included anti-SFTSV NP polyclonal antibody (α-SNP) [^8^](#_ENREF_8), anti-SFTSV NSs polyclonal antibody (α-SNSs) [^1^](#_ENREF_1), and anti-SFTSV Gn polyclonal antibody (α-SGn). The fluorescein isothiocyanate (FITC) conjugated sheep anti-mouse IgG antibody, FITC conjugated sheep anti-human IgG antibody, and the donkey polyclonal secondary antibody to rabbit IgG (Alexa Fluor® 555) were used as the secondary antibody for immunofluorescence assays (IFA) (Abcam). Inerferon-β (IFN-β) was purchased from Pepro Tech, Inc.

**Ticks collection and classification**

During April to May in 2014 when the peak activities of adult ticks occurred in Northern Xinjiang Province, China, the unfed adult ticks were collected by flagging in vegetation of Guertu County, Wusu City. Ticks were transported to laboratory alive and stored at -80 ºC before further examination. These ticks were examined under the microscope by an experienced technician based on the morphological characteristics and were classified as *Dermacentor nuttalli* [^9^](#_ENREF_9). To confirm the morphological classification, partial sequence of mitochondrial 16S rRNA from one tick was amplified by PCR as described [^10^](#_ENREF_10). The PCR products (454bp) were sequenced and analysed by Blastn comparison and phylogenetic analysis. The partial sequence of mitochondrial 16S rRNA was deposited in GenBank under the accession number MG921586.

**Roche 454 sequencing of ticks.**

Each tick pool was homogenized on ice in 3 ml DMEM supplemented with 2% FBS, followed by centrifuge (4500rpm, 5min) at 4°C to remove the debris. The clarified supernatants were treated with Turbo DNase (20 U/ml; Ambion), Benzonase (200 U/ml; NEB) and RNase A (0.1 mg/ml; Fermentas) at 37°C for 90 min to digest exogenous nucleic acids. The total viral RNA was extracted using a QIAamp Viral RNA Mini Kit (Qiagen, Dusseldorf, Germany) and eluted into 50 μl buffer AVE according to the manufacture’s manual. A volume of 13 μl total RNA from each pool was subjected to first- and second-strand cDNA synthesis with SuperScript III reverse transcriptase (Invitrogen) and Klenow fragment (Takara), respectively. Random primers with adapters designed according to the manufacturer`s instruction of Roche 454 GS FLX were used in both assays. PCR amplification was performed with the reaction products as templates and primers of adapter using FastStart™ High Fidelity PCR System (Roche) according to the manufacture’s instruction. PCR products were purified and applied to the Bioanalyzer 2100 instrument for library quantization. Then the products were subjected for pyrosequencing according to the manufacturer`s instruction. Reads collected from pyrosequencing were preprocessed by trimming primers and adaptors, and length filtering. The host-subtracted reads were assembled using Newbler assembler (454, v2.6). Contigs were annotated by searching the GenBank database using Blastn and Blastx comparisons.

**GTV isolation in Vero cells and identification by RT-PCR**

To isolate GTV from tick homogenates, clarified supernatants were diluted with DMEM with 2% FBS. Vero cells were seeded in 35 mm dishes one night before and incubated with two different dilutions respectively (v/v=1:4 and 1:40, 1ml for each incubation) at 37 ºC for 1.5 h. The supernatants were replaced with 2 ml fresh medium and maintained at 37 ºC for 3-4 days. Subsequently, the cells and culture supernatants from one dish were transferred to a T25 flask and further incubated with 3 ml fresh medium (5 ml culture supernatants in total) for another 3-4 days until the cell confluence reached over 90%. Then, 3 ml culture supernatants from the first passage were harvested, centrifuged to remove cell debris, sub-packaged and stored at -80 ºC. 100μl clarified culture supernatants were used for RNA purification and GTV detection by RT-PCR. The cells from the first passage were suspended in 3ml fresh medium. 1.7 ml cell suspension was discarded; leaving 1 ml in the flask incubated with the other 2 ml culture supernatants at 37 ºC for 1.5 h. Then 2ml fresh medium was added and the cells were maintained for 5-7 days as the second passage. 0.3 ml cell suspension was transferred to a 35mm dish added with 1.5 fresh medium. IFA was performed with this dish the next day to survey the virus infection. Virus passages were conducted for the following passages in the same way until infection was identified by IFA in over 50% of cells. Then the culture supernatants (5 ml) from that passage were harvested and clarified by centrifuge. 1 ml clarified culture supernatants were used to infect healthy Vero cells in a new T25 flask with 4ml fresh medium for the subsequent passages.

GTV RNA in culture supernatants from each passage was determined by RT-PCR. Total RNAs were purified from 100μl clarified culture supernatants from each passage using TRizol (Invitrogen) and transcribed into cDNA in 25μl volume with random primers (Promega) using M-MLV (Invitrogen) according to the manufacture’s manual. Then PCR was performed using the 2×Rapid Taq Master Mix (Vazyme Biotech) in 50μl volume according to the manufacture’s instruction with 2μl cDNA templates and the primers (C1F: 5’-ATGGGTGTTGTAGGAAAGATAG-3’ and C1R: 5’-GATTCCCTTTAACATTAACCTGG-3’) to amplify a fragment of M segment (2404-2983 nt, 580 bp in length). 10μl PCR products from each reaction were analyzed by 1% gel electrophoresis.

**Detection of GTV in tick pools and quantitative real-time RT-PCR of GTV in sera from mice**

Total RNA from equivalent supernatants of each tick pools (300μl) were extracted transcribed into cDNA as mentioned above, and used as the template for subsequent PCR detections using the 2×Rapid Taq Master Mix (Vazyme Biotech). Nested PCR was performed to amplify partial sequences of S segment with the primers C9F1: 5’-TTGCCATAGGGAATATGCTTC-3’ and C9R1: 5’-TGGGAGGTGATGATTGGATG-3’ for the first round (1180-1568 nt, 389 bp in length), and the primers C9F2: 5’-GGAGGTGATGATTGGATGA-3’ and C9R2: 5’-TTCTGGTGGGAAATTAGACAC-3’ for the second round (1272-1567 nt, 294 bp in length). PCR products were sequenced for further analysis.

To detect GTV RNA load in mice sera, total RNAs were purified from 10 μl sera using RNeasy Mini Kit (Qiagen, Hilden, Germany). The RNA standards were generated using the plasmid pT-Easy-GTV S^1065-1576^ as DNA template by *in vitro* transcription assay using a Transcript Aid T7 high yield transcription kit (Thermo Fisher Scientific) according to the manufacturer’s instruction. Partial S segment (1372-1528 nt) was amplified to determine GTV RNA copies (157bp in length) by real-time RT-PCR in a volume of 20μl mixture with primers: GTV-SF: 5’-ACTCTGAGCCACCCTGAC-3’ and GTV-SR: 5’- TGTATTTGCCCTCACTCG-3’ using the AceQ qPCR SYBR Green Master Mix (Vazyme) according to the manufacturer’s instruction.

**Cross-neutralization assays.** The serum samples were collected from mice challenged by GTV or SFTSV challenged mice on day 14 post challenge. Vero cells were seed into 96-well plates one night before. Each serum sample was serially diluted and mixed with an equal volume of 100 TCID_50_ viruses. Equal volumes of culture median without virus dilution and 100 TCID_50_ viruses were mixed as control. The mixtures were incubated as 37 ºC for 1.5h, then was added to the 96-well plates in quadruplicate. The plates were incubated at 37 ºC for 5 days, and virus infection was detected by IFA using α-SNP. The end-point titer was expressed as the reciprocal of the highest dilution that infection was not visualized.

**Supplementary Figures and Tables**

**Figure S1. Phylogeny of *Dermacentor nuttalli* from Guertu County.** Phylogenetic trees were constructed based on the partial sequence of mitochondria 16S rRNA using Maximum Likehood (ML) method and tested by bootstrap method of 1000 replicates. The sequence of the tick from Guertu County was indicated in bold and deposited in GenBank under accession number MG921586.

**Figure S2. RT-PCR detection of GTV RNA in cell culture supernatants of each passage.** Clarified supernatants from tick homogenates were diluted using DMEM supplemented with 2%FBS. Vero cells were incubated with the dilution of 1:4 and 1:40 (v/v) respectively. Blind passages were performed for both dilutions. RT-PCR was used to detect GTV generation in equivalent supernatants (100μl) from each passage.

**Figure S3. The alignments of the untranslational regions (UTRs) of L (A), M (B), and S (C) segments from GTV, SFTSV, and HRTV.** The nucleotides conserved in three sequences were shown in white characters and shaded in red. UTR, un-translational region; IUTR, the internal un-translational region in S segments flanked by NSs and NP ORFs.

**Figure S4. Sequence alignments of RNA-dependent RNA polymerase (RdRp), glycoproteins (G), nucleoproteins (NP), and nonstructural proteins (NSs).** Residues highlighted by which characters and red background are partially conserved residues are highlighted in red characters. Both of them are boxed with blue lines. (A) Sequence alignment of GTV, SFTSV, and HRTV RdRp. The functional domains were indicated by solid lines in accordance with the amino acid positions. Important functional motifs were shaded in light grey boxes. (B) Sequence alignment of GTV, SFTSV, and HRTV glycoproteins. Signal peptides (SP) and transmembrane regions (TM1, TM2, and TM3) were marked by cyan- and green-filled boxes, respectively. N-glycosylation sites were indicated by blue triangles. Disulfide bonds observed in crystal structures are numbered below the sequences in green for SFTSV Gn and blue for SFTSV Gc. Two cysteines important for SFTSV Gn stability are labeled with black asterisks and other four cysteines responsible for SFTSV Gn dimerization are labeled with purple asterisks. The neutralizing antibody epitope (α6) in SFTSV Gn are boxed in red. Two loops (Loop 1 and Loop 2) important for fusion and found in SFTSV Gc are boxed in black. (C) Sequence alignment of GTV, SFTSV, and HRTV NPs. The cyan asterisks labeled the residues responsible for oligmerization and purple asterisks indicated the amino acids important for RNA binding observed in SFTSV NP crystal structure. The residues that are conserved in GTV and SFTSV NP but not in HRTV NP are shaded in green. (D) Sequence alignment of GTV, SFTSV, and HRTV NSs. The conserved PXXP motifs are shaded in green.

**Figure S5. The reporter assays using SFTSV replicon system with the substitution of SFTSV, GTV, or HRTV NP, respectively.** (A) The reporter assays were performed using the system with the *luciferase* reporter plasmids. The relative luciferase activity (Rel. Luc. Act.) in each groups with NP substitution was measured in triplicates. (B) The fold changes of luciferase activities from each group as indicated in (A) were shown as the luciferase activities of other groups expressed as the levels normalized to the SFTSV group. The activities from the SFTSV NP group were set as 100%. The data were shown as means ±SD.

**Figure S6. The ML phylogenetic tree built based on the partial sequences of S segments from GTV, SFTSV, and HRTV.** Sequences of PCR products from four GTV positive tick groups were included. All strains were presented as “virus name strain”. The GTV strains were shown in bold characters and the isolated virus strain DXM was labeled by black solid circle. The ML tree was constructed using Mega 5.0 and tested by the bootstrap method with 1000 replications.

**Figure S7. Immunofluorescence assays of human serum samples against GTV infected cells.** Vero cells were seeded in 96-well plates and infected by GTV. The infected cells were blotted by IFA at 48 h p.i. using human serum samples in the 20-fold dilution. Images were taken under the exposure time of 1000ms, and a few were presented in this figure. The first panel showed the images taken from positive controls using α-SGn, α-SNP, and serum samples collected from GTV and SFTSV challenged mice (α-GTV and α-SFTSV) on day 14 respectively as the primary antibodies. The second panel showed the images taken from three serum samples identified to be neutralization antibodies (H3, H5, and F3) against GTV and one image from H3 blotted healthy cells (Negative). The third panel showed the images taken from four other serum samples of antibody against GTV antigen but with no neutralizing activity (H97, H54, F7, and F113). The forth panel showed the images of four serum samples from Hubei Province which presented negative responses to GTV antigens. H, the serum samples from herdsmen; F, the serum samples from famers; HB, the serum samples from Hubei Province.

**Table S1**. The length of segments and the nucleotide sequence identities of GTV S, M, and L segments in comparison with other phleboviruses

|  | GTV | SFTSV | HRTV | HIGV | BHAV | UUKV | RVFV |
| --- | --- | --- | --- | --- | --- | --- | --- |
| L | 6366 | 6368 (76%) | 6368 (67%) | 6368 (63%) | 6333 (44%) | 6423 (45%) | 6404 (43%) |
| M | 3371 | 3378 (72%) | 3427 (59%) | 3328 (55%) | 3304 (41%) | 3229 (36%) | 3885 (32%) |
| S | 1744 | 1744 (74%) | 1772 (60%) | 1694 (51%) | 1870 (32%) | 1720 (29%) | 1690 (39%) |

The % nucleotide sequence identity to GTV is shown in parentheses.

GTV, Guertu virus; SFTSV, severe fever with thrombocytopenia syndrome virus; HRTV, Heartland virus; HIGV, Hunter Island Group virus; BHAV, Bhanja virus; UUKV, Uukuniemi virus; RVFV, Rift Valley fever virus.

**Table S2** Nucleotide and amino acid pairwise sequence identity values for GTV, SFTSV, and HRTV

|  | % nucleotide or amino acid sequence identity | | | | | | | | | | | | | | |
| --- | --- | --- | --- | --- | --- | --- | --- | --- | --- | --- | --- | --- | --- | --- | --- |
|  | RdRp | | |  | G | | |  | NP | | |  | NSs | | |
|  | GTV | SFTSV | HRTV |  | GTV | SFTSV | HRTV |  | GTV | SFTSV | HRTV |  | GTV | SFTSV | HRTV |
| GTV | - | 76 | 67 |  | - | 71 | 60 |  | - | 78 | 64 |  | - | 68 | 58 |
| SFTSV | 89 | - | 68 |  | 78 | - | 61 |  | 88 | - | 64 |  | 74 | - | 57 |
| HRTV | 73 | 73 | - |  | 60 | 61 | - |  | 60 | 61 | - |  | 59 | 59 | - |

The pairwise comparison of nucleotide identities were shown on upper right, and the amino acid identities were shown on lower left.

**Table S3** The end-point titers assayed by cross-neutralization tests

| Serum samples | Viruses (100 TCID_50_ per well) | |
| --- | --- | --- |
|  | GTV | SFTSV |
| GTV infected mice | 2^5^ | 2^6^ |
| SFTSV infected mice | 2^4^ | 2^6^ |

The end-point titers were shown as the reciprocal of the highest dilution of serum samples to prevent the infection.

**References**

1 Ning, Y. J. *et al.* Viral suppression of innate immunity via spatial isolation of TBK1/IKKepsilon from mitochondrial antiviral platform. *J Mol Cell Biol* **6**, 324-337, doi:10.1093/jmcb/mju015 (2014).

2 Zhang, Y. *et al.* Isolation, characterization, and phylogenic analysis of three new severe fever with thrombocytopenia syndrome bunyavirus strains derived from Hubei Province, China. *Virologica Sinica* **32**, 89-96, doi:10.1007/s12250-017-3953-3 (2017).

3 McMullan, L. K. *et al.* A new phlebovirus associated with severe febrile illness in Missouri. *The New England journal of medicine* **367**, 834-841, doi:10.1056/NEJMoa1203378 (2012).

4 Flick, R. & Pettersson, R. F. Reverse genetics system for Uukuniemi virus (Bunyaviridae): RNA polymerase I-catalyzed expression of chimeric viral RNAs. *J Virol* **75**, 1643-1655, doi:10.1128/JVI.75.4.1643-1655.2001 (2001).

5 Ning, Y. J. *et al.* Heartland virus NSs protein disrupts host defenses by blocking the TBK1 kinase-IRF3 transcription factor interaction and signaling required for interferon induction. *J Biol Chem*, doi:10.1074/jbc.M117.805127 (2017).

6 Lam, T. T. *et al.* Evolutionary and molecular analysis of the emergent severe fever with thrombocytopenia syndrome virus. *Epidemics* **5**, 1-10, doi:10.1016/j.epidem.2012.09.002 S1755-4365(12)00046-1 [pii] (2013).

7 Huang, D. *et al.* A Cluster of Symptomatic and Asymptomatic Infections of Severe Fever with Thrombocytopenia Syndrome Caused by Person-to-Person Transmission. *Am J Trop Med Hyg* **97**, 396-402, doi:10.4269/ajtmh.17-0059 (2017).

8 Huang, D. *et al.* A Cluster of Symptomatic and Asymptomatic Infections of Severe Fever with Thrombocytopenia Syndrome Caused by Person-to-Person Transmission. *The American journal of tropical medicine and hygiene*, doi:10.4269/ajtmh.17-0059 (2017).

9 Yu, X. & Ye, R. The ticks fauna of Xinjiang. (1997).

10 Mangold, A. J., Bargues, M. D. & Mas-Coma, S. Mitochondrial 16S rDNA sequences and phylogenetic relationships of species of Rhipicephalus and other tick genera among Metastriata (Acari: Ixodidae). *Parasitology research* **84**, 478-484 (1998).
