# Supplementary figures and images for: A novel tick-borne phlebovirus, closely related to severe fever with thrombocytopenia syndrome virus and Heartland virus, is a potential pathogen
Source: Emerg Microbes Infect. 2018 May 25;7:95. doi: 10.1038/s41426-018-0093-2 (PMC5970217; doi:10.1038/s41426-018-0093-2)

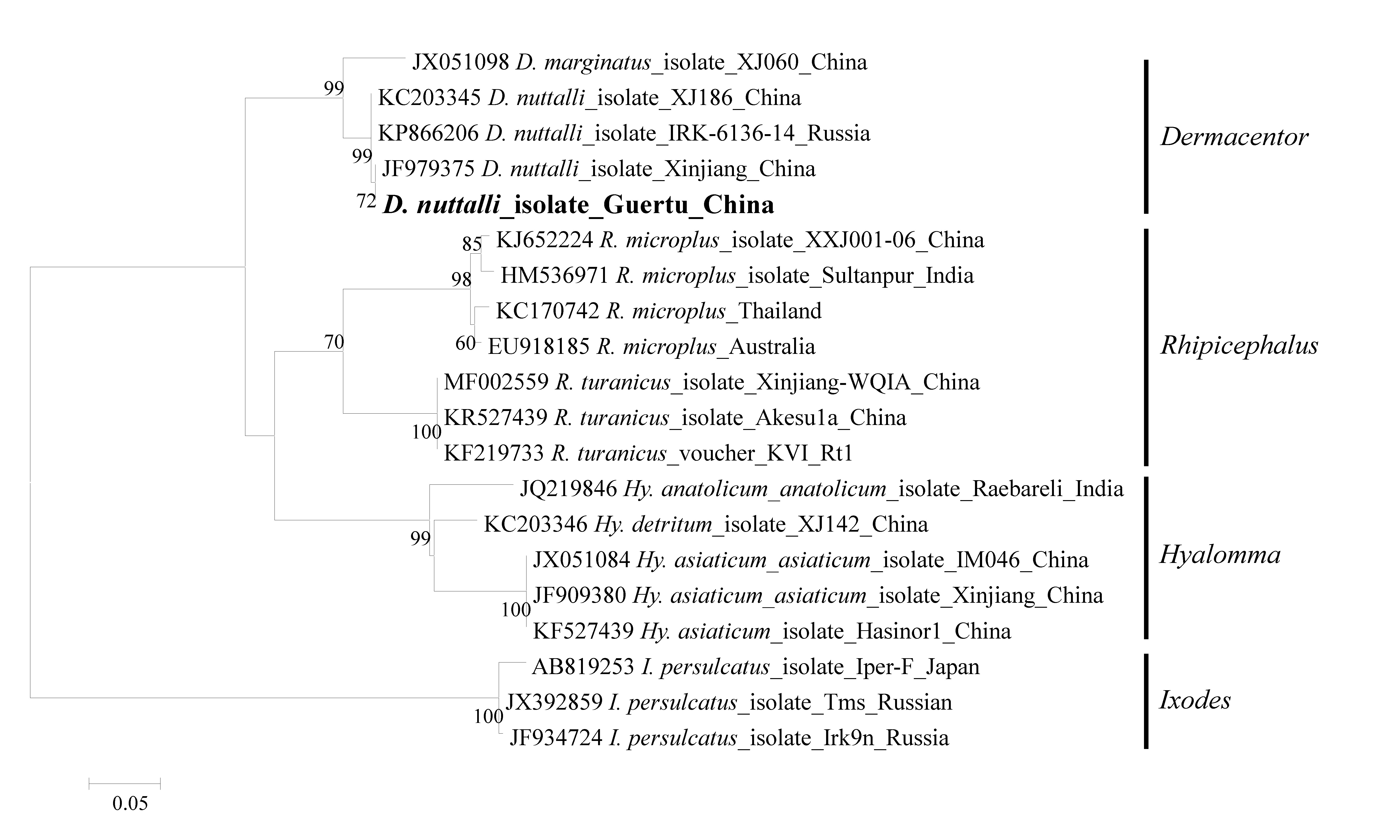

Supplement: Supplementary file 4 — Figure S3 [file 41426_2018_93_MOESM4_ESM.jpg]

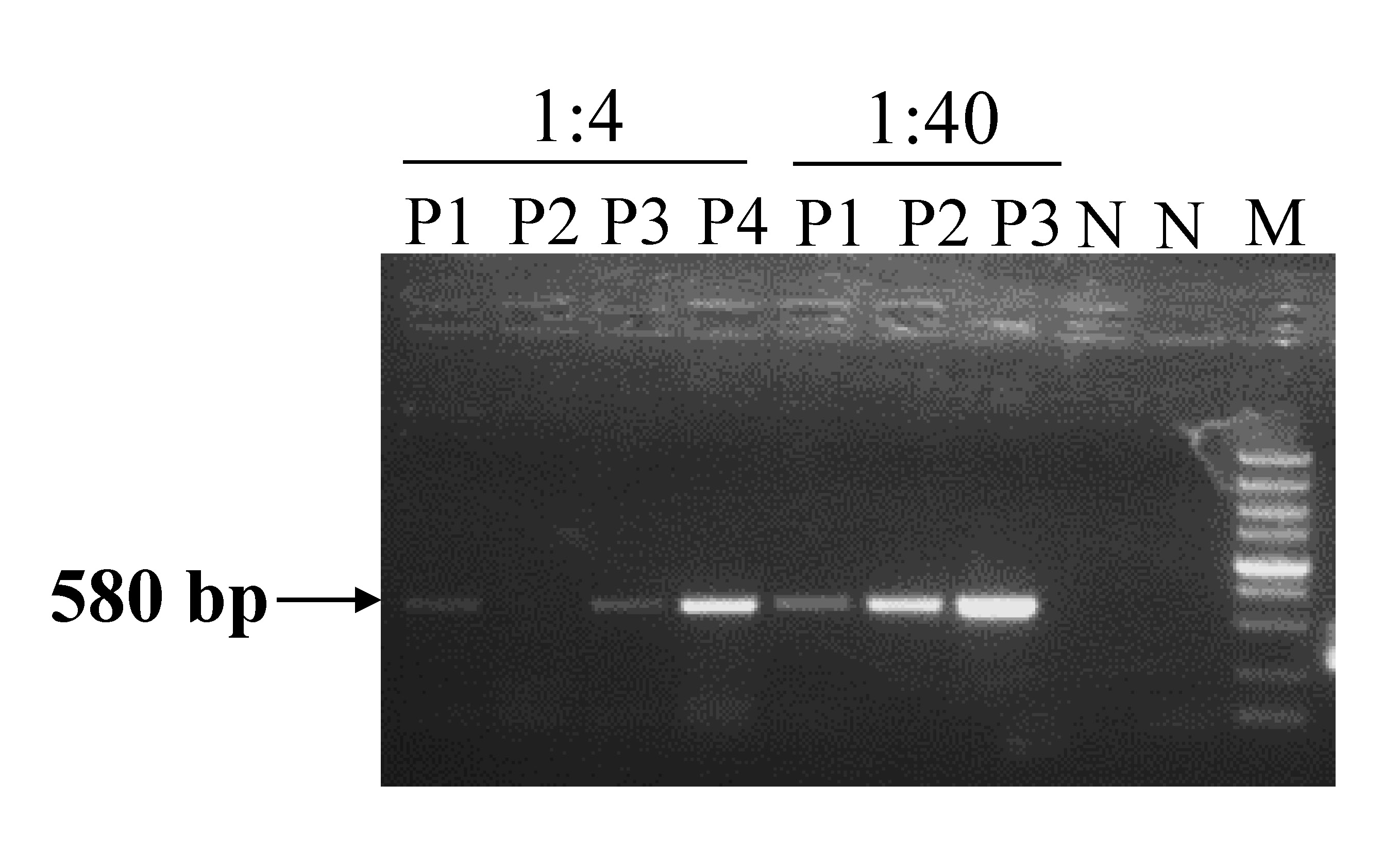

Supplement: Supplementary file 5 — Figure S4 [file 41426_2018_93_MOESM5_ESM.jpg]

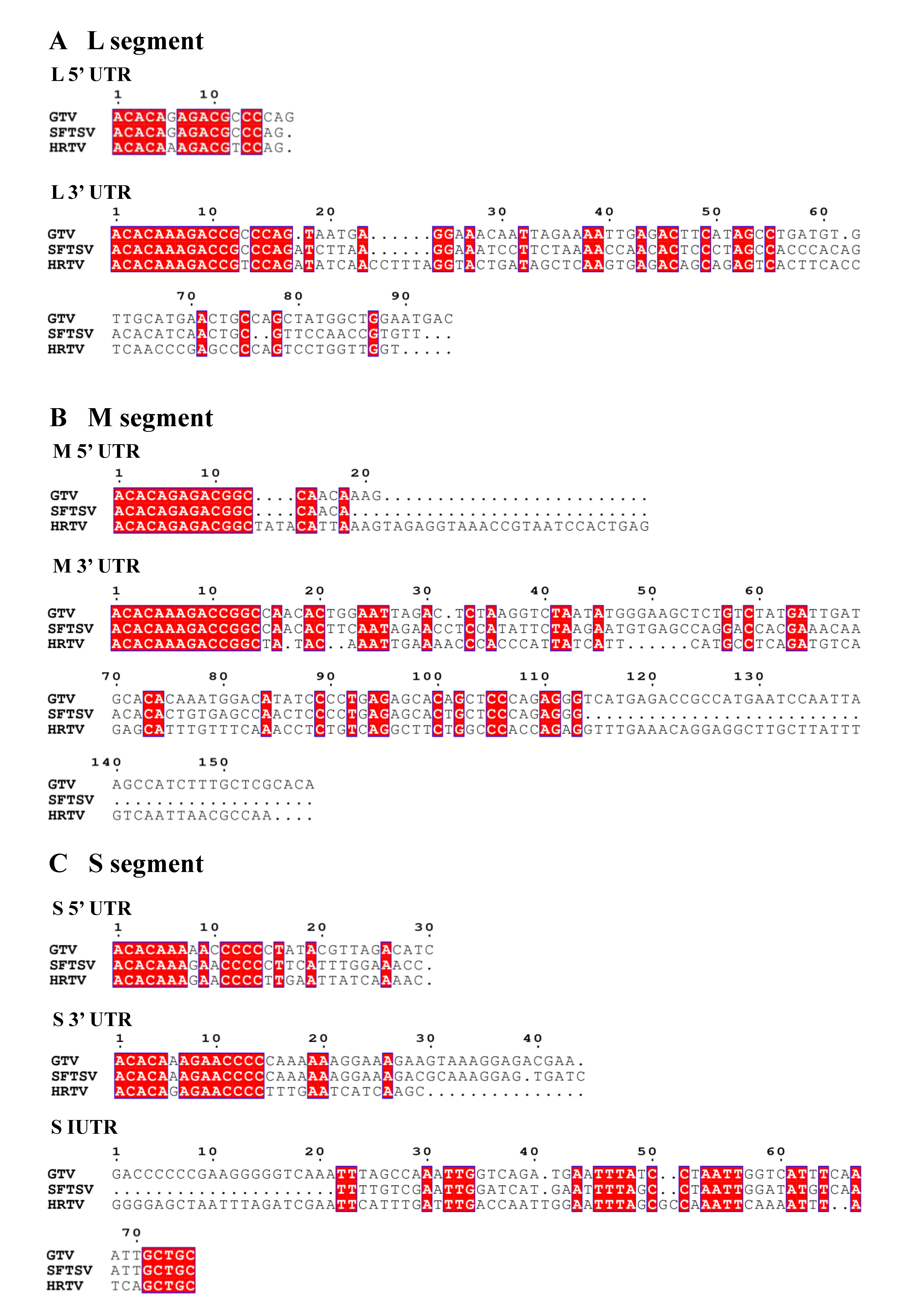

Supplement: Supplementary file 6 — Figure S5 [file 41426_2018_93_MOESM6_ESM.jpg]

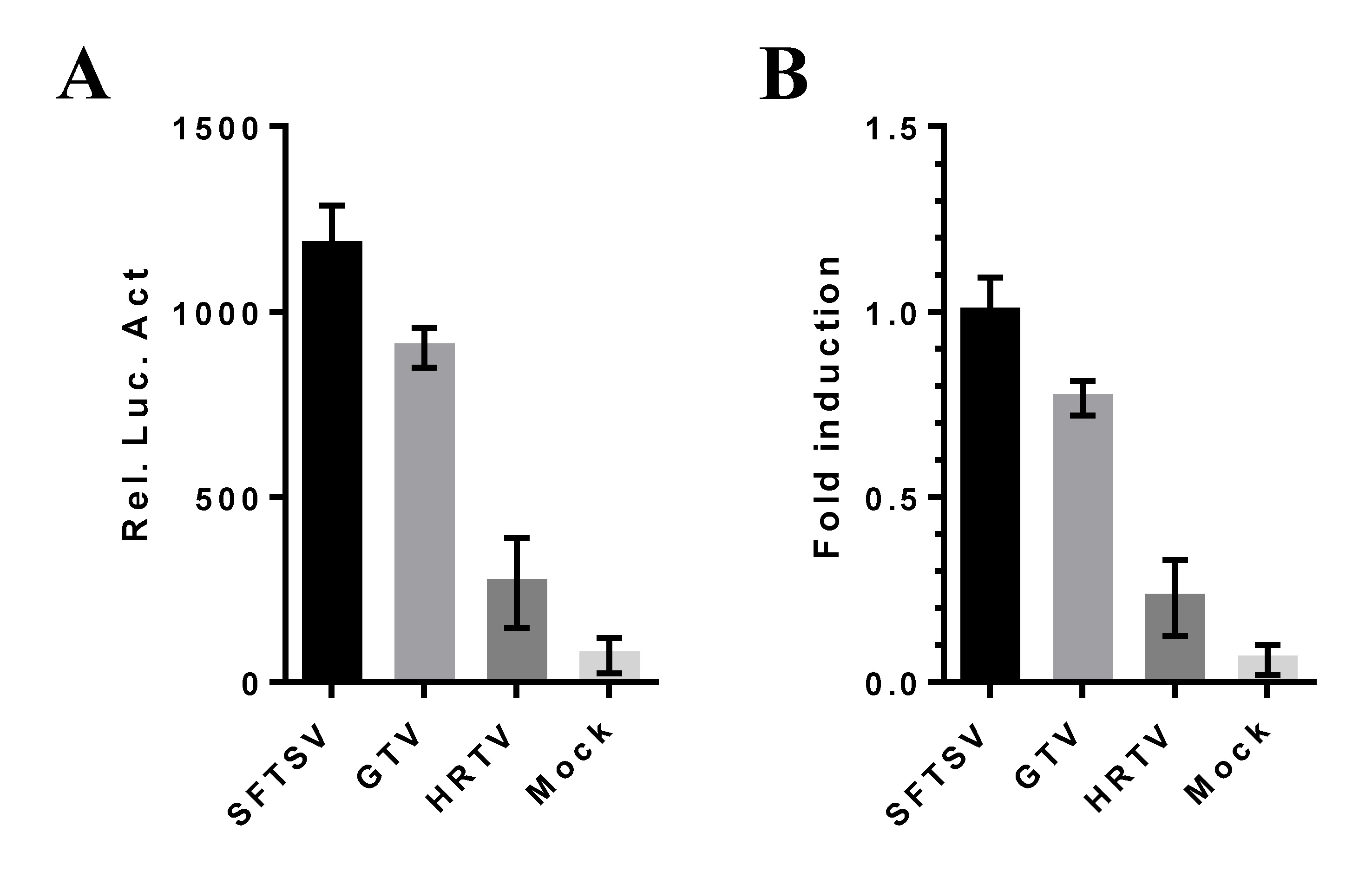

Supplement: Supplementary file 8 — Figure S7 revision [file 41426_2018_93_MOESM8_ESM.jpg]

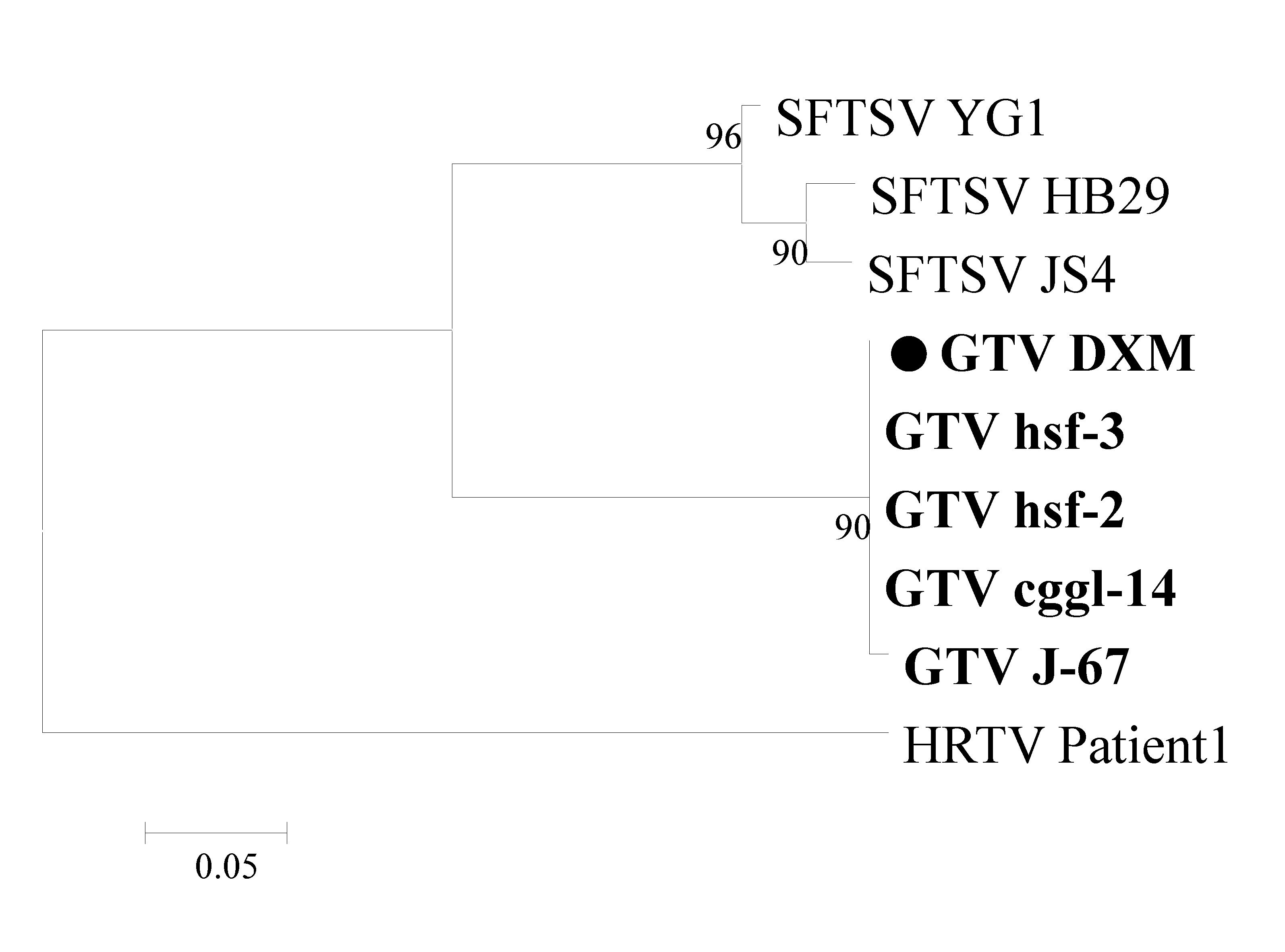

Supplement: Supplementary file 9 — Figure S8 revision [file 41426_2018_93_MOESM9_ESM.jpg]

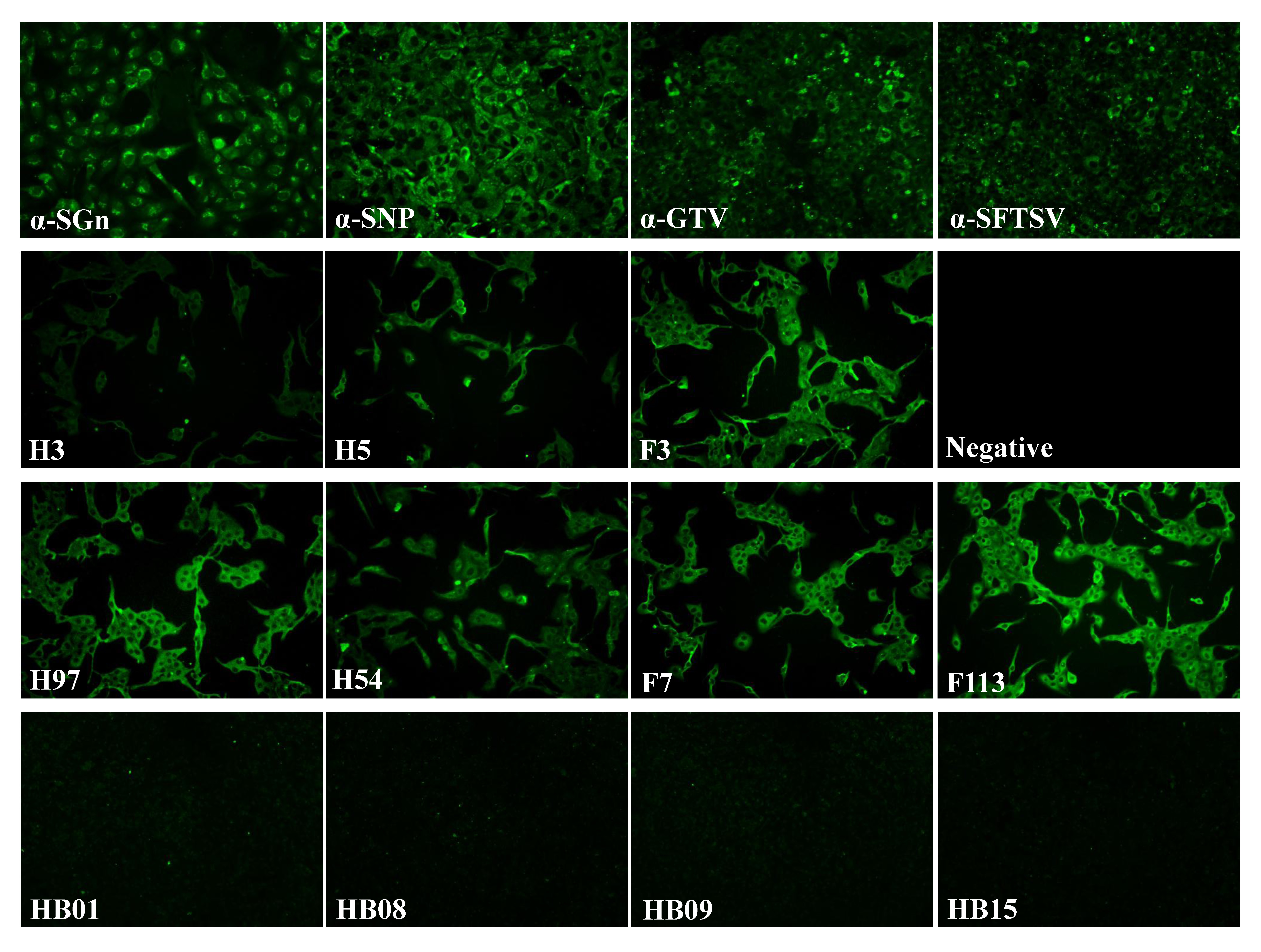

Supplement: Supplementary file 10 — Figure S9 revision [file 41426_2018_93_MOESM10_ESM.jpg]
