# Supplementary material for: A novel tick-borne phlebovirus, closely related to severe fever with thrombocytopenia syndrome virus and Heartland virus, is a potential pathogen
Source: Emerg Microbes Infect. 2018 May 25;7:95. doi: 10.1038/s41426-018-0093-2 (PMC5970217; doi:10.1038/s41426-018-0093-2)
Supplement: Supplementary file 7 — Figure S6 [file 41426_2018_93_MOESM7_ESM.pdf]

Figure S4 A

```
         1        10        20        30        40        50        60        70
GTV     MNLEVLCGRINAEDGLSLGEPGLFDQIYDRPGLPDLDVSVDATGVVVDIGAIPDSASHLGSSINAGVINI
SFTSV   MNLEVLCGRINVENGLSLGEPGLYDQIYDRPGLPDLDVTVDATGVTVDIGAVPDSASQLGSSINAGLITI
HRTV    MNLEALCSRVLSERGLSTGEPGVYDQIFERPGLPNLEVTVDSTGVVVDVGAIPDSASQLGSSINAGVLTI
```

Endonuclease

```
                 80        90       100       110       120       130       140
GTV     QLSEAYKINHDFTFSGLSKTTDRRLSEVFPIAHDGSDGMTPDVIHTRLDGTIVVVEFTTTRSLNMGGLEA
SFTSV   QLSEAYKINHDFTFSGLSKTTDRRLSEVFPITHDGSDGMTPDVIHTRLDGTIVVVEFSTTRSHNIGGLEA
HRTV    PLSEAYKINHDFTFSGLTKTTDRKLSEVFPLVHDGSDSMTPDVIHTRLDGTVVVIEFTTTRSTNMGGLEA
```

Endonuclease

```
                150       160       170       180       190       200       210
GTV     AYRTKVEKYRDPISRRVDLMANARVFYGIIVVSSSGVLTNMPLTQDEAEELMYRFCIANEIYTKARTMDA
SFTSV   AYRTKIEKYRDPISRRVDIMENPRVFFGVIVVSGGVLSNMPLTQDEAEELMYRFCIANEIYTKARSMDA
HRTV    AYRSKLEKYRDPLNRRTDIMPDASIYFGIIVVSASGVLTNMPLTQDEAEELMFRFCVANEIYSQARAMDA
```

DUF3770 domain

```
                220       230       240       250       260       270       280
GTV     EVELQKTEEELEAVSRALSFFGLFDPNLQKVEETFPNSDIRMLEEFLSSPADVKFITDTLKAKEIESYEE
SFTSV   DIELQKSEEELEAISRALSFFSLFEPNIERVEGTFPNSEIEMLEQFLSTPADVDFITKTLKAKEVEAYAD
HRTV    EVELQKSEEEYEAISRARAFFTLFDYDDGKLSEAFPNSDIEMLRRFLSQPVDTSFVTTTLKEKEQEAYKR
```

DUF3770 domain

```
                290       300       310       320       330       340       350
GTV     LCHTHFLKPEKTMQERLELNRCDAINKTQDLLAGLHQQSNKQTSINKGTVKLPPWLPKPSNESIDIKTDS
SFTSV   LCDSHYLKPEKTIQERLEINRCEAIDKTQDLLAGLHARSNKQTSLNRGTVKLPPWLPKPSSESIDIKTDS
HRTV    MCEEHYLKSGMSTKERLEANRSDAIDKTRALMERLHNMSSKELHSNKSTVKLPPWVVKPSDRTLDVKTDT
```

DUF3770 domain

```
                360       370       380       390       400       410       420
GTV     GFGSLMDHGPYGELWAKCLLDVSLGNVEGVVSDPAKELGIAVSDDPEMDTPKEAKITYRRFRPTLSLGAK
SFTSV   GFGSLMDHGAYGELWAKCLLDVSLGNVEGVVSDPAKELDIAISDDPEKDTPKEAKITYRRFKPALSSSAR
HRTV    GSGELLNHGPYGELWSRCFLEIVLGNVEGVISSPEKELEIAISDDPEADTPKAAKIKYHRFRPELSLESK
```

DUF3770 domain

```
                430       440       450       460       470       480       490
GTV     QEFSLQGVEGKKWKRSAANMKKEKESHESLSPYLDVDDISDFLTFNNLLSESRYGDESIQRAVTILLEKA
SFTSV   QEFSLQGVEGKKWKRMAANQKKEKESHETLSPFLDVEDIGDFLTFNNLLADSRYGDESVQRAVSILLEKA
HRTV    HEFSLQGIEGKRWKHSARNVLKDEMSHKTMSPFVDVSNIEEFLIMNNLLNDTSFNREGLQETINLLLEKA
```

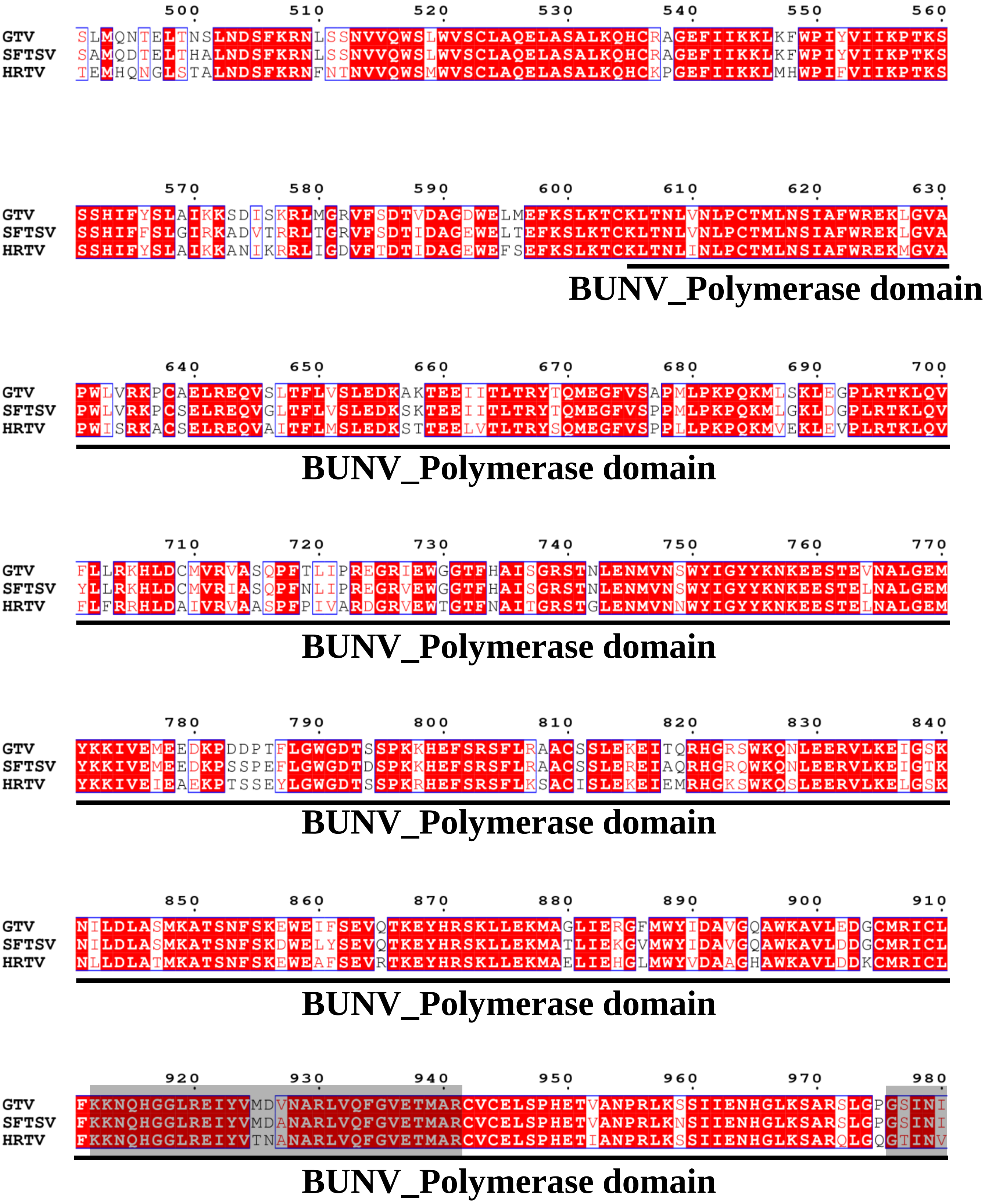

500 510 520 530 540 550 560
GTV SLMQNTELTNSLNDSFKRNLSSNVVQWSLWVSCLAQELASALKQHCRAGEFIIKKLKFWPIYVIIKPTKS
SFTSV SAMQDTELTHALNDSFKRNLSSNVVQWSLWVSCLAQELASALKQHCRAGEFIIKKLKFWPIYVIIKPTKS
HRTV TEMHQNGLSTALNDSFKRNFNTNVVQWSMWVSCLAQELASALKQHCKPGEFIIKKLMHWPIFVIIKPTKS
570 580 590 600 610 620 630
GTV SSHIFYSLAIKKSDISKRLMGRVFSDTVDAGDWELMEFKSLKTCKLTNLVNLPCTMLNSIAFWREKLGVA
SFTSV SSHIFFSLGIRKADVTRRLTGRVFSDTIDAGEWELTEFKSLKTCKLTNLVNLPCTMLNSIAFWREKLGVA
HRTV SSHIFYSLAIKKANIKRRLIGDVFTDTIDAGEWEFSEFKSLKTCKLTNLINLPCTMLNSIAFWREKMGVA
BUNV_Polymerase domain
640 650 660 670 680 690 700
GTV PWLVRKPCAELREQVSLTFLVSLEDKAKTEEIITLTRYTQMEGFVSAPMLPKPQKMLSKLEGPLRTKLQV
SFTSV PWLVRKPCSELREQVGLTFLVSLEDKSKTEEIITLTRYTQMEGFVSPPMLPKPQKMLGKLDGPLRTKLQV
HRTV PWISRKACSELREQVAITFLMSLEDKSTTEELVTLTRYSQMEGFVSPPLLPKPQKMVEKLEVPLRTKLQV
BUNV_Polymerase domain
710 720 730 740 750 760 770
GTV FLLRKHLDCMVRVASQPFTLIPREGRIEWGGTFHAISGRSTNLENMVNSWYIGYYKNKEESTEVNALGEM
SFTSV YLLRKHLDCMVRIASQPFNLIPREGRVEWGGTFHAISGRSTNLENMVNSWYIGYYKNKEESTELNALGEM
HRTV FLFRRHLDAIVRVAASPFPIVARDGRVEWTGTFNAITGRSTGLENMVNNWYIGYYKNKEESTELNALGEM
BUNV_Polymerase domain
780 790 800 810 820 830 840
GTV YKKIVEMEEDKPDDPTFLGWGDTSSPKKHEFSRSFLRAACSSLEKEITQRHGRSWKQNLEERVLKEIGSK
SFTSV YKKIVEMEEDKPSSPEFLGWGDTDSPKKHEFSRSFLRAACSSLEREIAQRHGRQWKQNLEERVLKEIGTK
HRTV YKKIVEIEAEKPTSSEYLGWGDTSSPKRHEFSRSFLKSACISLEKEIEMRHGKSWKQSLEERVLKELGSK
BUNV_Polymerase domain
850 860 870 880 890 900 910
GTV NILDLASMKATSNFSKEWEIFSEVQTKEYHRSKLLEKMAGLIERGFMWYIDAVGQAWKAVLEDGCMRICL
SFTSV NILDLASMKATSNFSKDWELYSEVQTKEYHRSKLLEKMATLIEKGVMWYIDAVGQAWKAVLDDGCMRICL
HRTV NLLDLATMKATSNFSKEWEAFSEVRTKEYHRSKLLEKMAELIEHGLMWYVDAAGHAWKAVLDDKCMRICL
BUNV_Polymerase domain
920 930 940 950 960 970 980
GTV FKKNQHGGLREIYVMDVNARLVQFGVETMARCVCELSPHETVANPRLKSSIIENHGLKSARSLGPGSINI
SFTSV FKKNQHGGLREIYVMDANARLVQFGVETMARCVCELSPHETVANPRLKNSIIENHGLKSARSLGPGSINI
HRTV FKKNQHGGLREIYVTNANARLVQFGVETMARCVCELSPHETIANPRLKSSIIENHGLKSARQLGQGTINV
BUNV_Polymerase domain

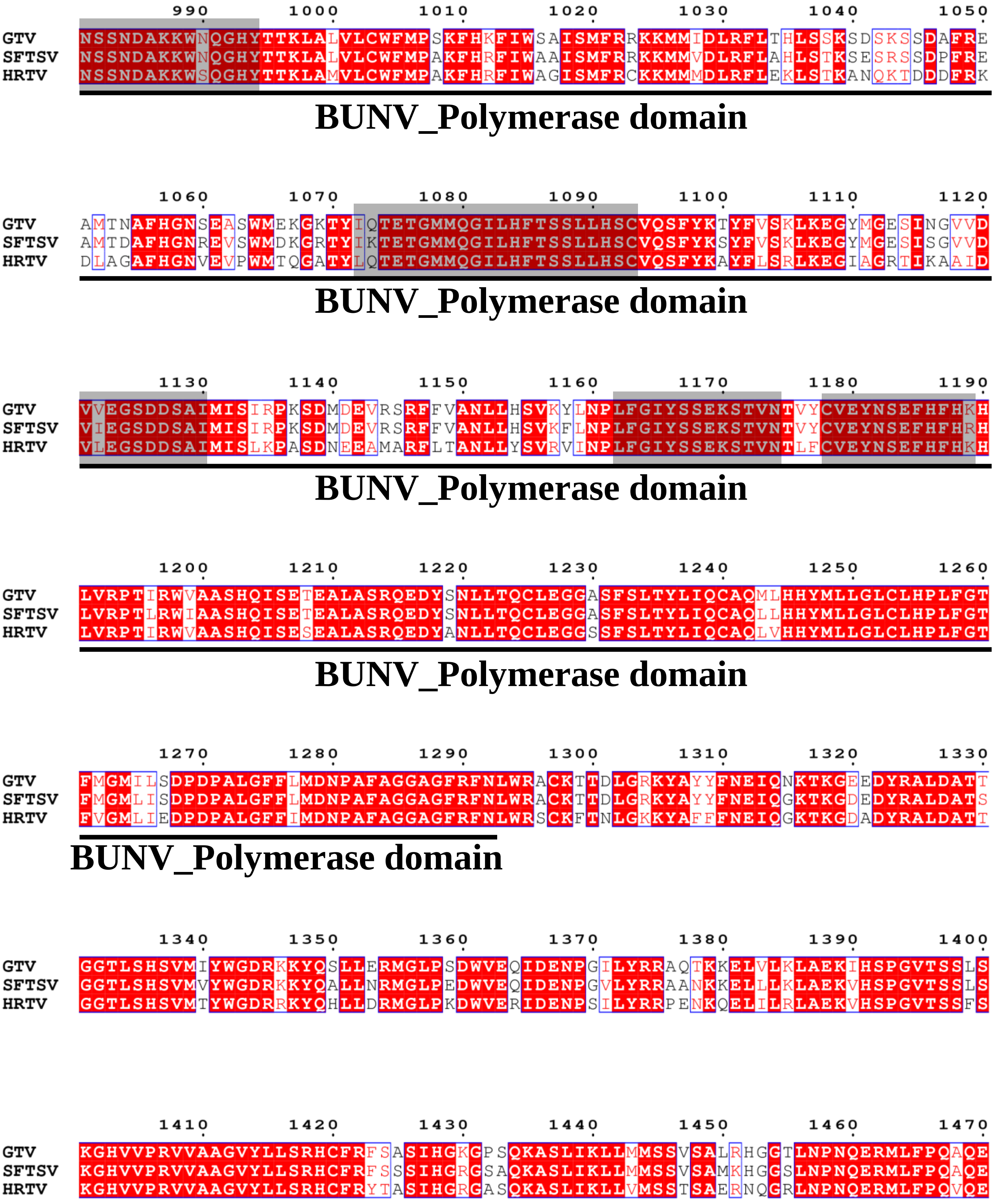
990 1000 1010 1020 1030 1040 1050
GTV NSSNDAKKWNQGHYTTKLALVLCWFMPSKFHKFIWSAISMFRRKKMMIDLRFLTHLSSKSDSKSSDAFRE
SFTSV NSSNDAKKWNQGHYTTKLALVLCWFMPAKFHRFIWAAISMFRRKKMMVDLRFLAHLSTKSESRSSDPFRE
HRTV NSSNDAKKWSQGHYTTKLAMVLCWFMPAKFHRFIWAGISMFRCKKMMMDLRFLEKLSTKANQKTDDDFRK
BUNV_Polymerase domain
1060 1070 1080 1090 1100 1110 1120
GTV AMTNAFHGNSEASWMEKGKTYIQTETGMMQGILHFTSSLLHSCVQSFYKTYFVSKLKEGYMGESINGVVD
SFTSV AMTDAFHGNREVSWMDKGRTYIKTETGMMQGILHFTSSLLHSCVQSFYKSYFVSKLKEGYMGESISGVVD
HRTV DLAGAFHGNVEVPWMTQGATYLQTETGMMQGILHFTSSLLHSCVQSFYKAYFLSRLKEGIAGRTIKAAID
BUNV_Polymerase domain
1130 1140 1150 1160 1170 1180 1190
GTV VVEGSDDSAIMISIRPKSDMDEVRSRFFVANLLHSVKYLNPLFGIYSSEKSTVNTVYCVEYNSEFHFHKH
SFTSV VIEGSDDSAIMISIRPKSDMDEVRSRFFVANLLHSVKFLNPLFGIYSSEKSTVNTVYCVEYNSEFHFHRH
HRTV VLEGSDDSAIMISLKPASDNEEAMARFLTANLLYSVRVINPLFGIYSSEKSTVNTLFCVEYNSEFHFHKH
BUNV_Polymerase domain
1200 1210 1220 1230 1240 1250 1260
GTV LVRPTIRWVAASHQISETEALASRQEDYSNLLTQCLEGGASFSLTYLIQCAQMLHHYMLLGLCLHPLFGT
SFTSV LVRPTLRWIAASHQISETEALASRQEDYSNLLTQCLEGGASFSLTYLIQCAQLLHHYMLLGLCLHPLFGT
HRTV LVRPTIRWVAASHQISESEALASRQEDYANLLTQCLEGGSSFSLTYLIQCAQLVHHYMLLGLCLHPLFGT
BUNV_Polymerase domain
1270 1280 1290 1300 1310 1320 1330
GTV FMGMILSDPDPALGFFLMDNPAFAGGAGFRFNLWRACKTTDLGRKYAYYFNEIQNKTKGEEDYRALDATT
SFTSV FMGMLISDPDPALGFFLMDNPAFAGGAGFRFNLWRACKTTDLGRKYAYYFNEIQGKTKGDEDYRALDATS
HRTV FVGMLIEDPDPALGFFIMDNPAFAGGAGFRFNLWRSCKFTNLGKKYAFFFNEIQGKTKGDADYRALDATT
BUNV_Polymerase domain
1340 1350 1360 1370 1380 1390 1400
GTV GGTLSHSVMIYWGDRKKYQSLLERMGLPSDWVEQIDENPGILYRRAQTKKELVLKLAEKIHSPGVTSSLS
SFTSV GGTLSHSVMVYWGDRKKYQALLNRMGLPEDWVEQIDENPGVLYRRAANKKELLLKLAEKVHSPGVTSSLS
HRTV GGTLSHSVMTYWGDRRKYQHLLDRMGLPKDWVERIDENPSILYRRPENKQELILRLAEKVHSPGVTSSFS
1410 1420 1430 1440 1450 1460 1470
GTV KGHVVPRVVAAGVYLLSRHCFRFSASIHGKGPSQKASLIKLLMMSSVSALRHGGTLNPNQERMLFPQAQE
SFTSV KGHVVPRVVAAGVYLLSRHCFRFSSSIHGRGSAQKASLIKLLMMSSVSAMKHGGSLNPNQERMLFPQAQE
HRTV KGHVVPRVVAAGVYLLSRHCFRYTASIHGRGASQKASLIKLLVMSSTSAERNQGRLNPNQERMLFPQVQE

```
                   1480      1490      1500      1510      1520      1530      1540
GTV     YERVCTLLEEVENLTGKFVVRERNIVRSRIDLFQDPVDLRCKAEDLVSEVWFGMKRTKLGPRLLKEEWDK
SFTSV   YDRVCTLLEEVEHLTGKFVVRERNIVRSRIDLFQEPVDLRCKAEDLVSEVWFGLKRTKLGPRLLKEEWDK
HRTV    YERVLTLLDEVTALTGKFVVRERNIVKSRVELFQEPVDLRCKAENLIAEMWFGLKRTKLGPRLLKEEWDK

                   1550      1560      1570      1580      1590      1600      1610
GTV     LRASFAWLSTDPAETLRDGPFMSHVQFRNFIAHVDAKSRSVRLLGAPVKKSGGVTTINQVVRMNFFPGFC
SFTSV   LRASFAWLSTDPSETLRDGPFLSHVQFRNFIAHVDAKSRSVRLLGAPVKKSGGVTTISQVVRMNFFPGFS
HRTV    LRASFSWLSTDHKETLDVGPFLSHVQFRNFIAHVDAKSRSVRLLGAPVKKSGGVTTVSQVVKSNFFPGFI

                   1620      1630      1640      1650      1660      1670      1680
GTV     LDAEKSLDNQERLESISILKHLLFMILNGPYTEEYKLDMIMEAFESLALPQPTEVIRKSRTMTLCLISNY
SFTSV   LEAEKSLDNQERLESISILKHVLFMVLNGPYTEEYKLEMIIEAFSTLVIPQPSEVIRKSRTMTLCLLSNY
HRTV    LDSSESLDDQERVEGVSILKHILFMTLNGPYTDEQKKAMVLETFQYFALPHAAEVVKRSRSLTLCLMKNF

                   1690      1700      1710      1720      1730      1740      1750
GTV     FSQKGGSILDQIEKAQSGTLGGFSKPQRTFTRPGGSIGYKGKGVWTGVMEDTHVQLLIDGDGTNNWLEEI
SFTSV   LSSKGGSILDQIERAQSGTLGGFSKPQKTFIRPGGVGYKGKGVWTGVMEDTHVQILIDGDGTSNWLEEI
HRTV    IEQRGGSILDQIEKAQSGTVGGFSKPQKPYRKQSGGIGYKGKGVWSGIMENTNVQILIDGDGSSNWIEEI

                   1760      1770      1780      1790      1800      1810      1820
GTV     RLSSEARLYDVIESIRRLCDDLGINNRVASSFRGHCMVRLSGFKIKPASRTDGCPIRVLERGFWIRELQN
SFTSV   RLSSDARLYDVIESIRRLCDDLGINNRVASAYRGHCMVRLSGFKIKPASRTDGCPVRIMERGFRIRELQN
HRTV    RLSSESRLFDVIESVRRLCDDINVNNRVTSSFRGHCMVRLSNFKVKPASRVEGCPVRLMPSSFRIKELQN

                   1830      1840      1850      1860      1870      1880      1890
GTV     PDEVFMRVRGDILNLSVRIQEGRIMNILSYRPRDTDISESAAAFLWSNKDSFSFGKKEPSCSWICLKTLD
SFTSV   PDEVKMRVRGDILNLSVTIQEGRVMNILSYRPRDTDISESAAAYLWSNRDLFSFGKKEPSCSWICLKTLD
HRTV    PDEVFLRVRGDILNLSILLQEDRVMNLLSYRARDTDISESAASYLWMNRTDFSFGKKEPSCSWMCLKTLD

                   1900      1910      1920      1930      1940      1950      1960
GTV     NWAWSHASTLLATKRKTPGINNELMGNIFRDCLEGSLRKQGLMRSKLSELVEKNVIPLTTQELVDILEED
SFTSV   NWAWSHASVLLANDRKTQGIDNRAMGNIFRDCLEGSLRKQGLMRSKLTEMVEKNVVPLTTQELVDILEED
HRTV    SWAWNQAARVLERNIKTPGIDNTAMGNIFKDCLESSLRKQGLLRSRIAEMVERHVIPLTSQELVDILEED
```

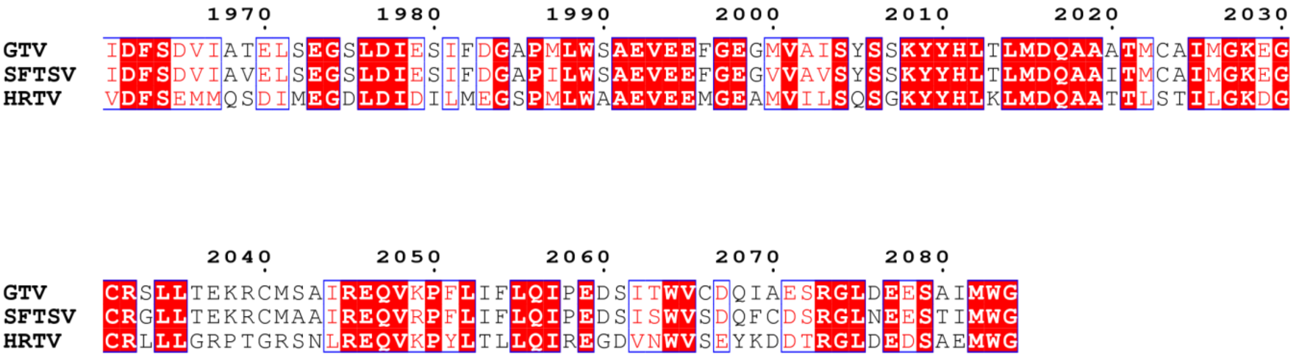
1970 1980 1990 2000 2010 2020 2030
GTV IDFSDVIATELSEGSLDIESIFDGAPMLWSAEVEEFGEGMVAISYSSKYYHLTLMDQAAATMCAIMGKEG
SFTSV IDFSDVIAVELSEGSLDIESIFDGAPILWSAEVEEFGEGVVAVSYSSKYYHLTLMDQAAITMCAIMGKEG
HRTV VDFSEMMQSDIMEGDLDIDILMEGSPMLWAAEVEEMGEAMVILSQSGKYYHLKLMDQAATTLSTILGKDG
2040 2050 2060 2070 2080
GTV CRSLLTEKRCMSAIREQVKPFLIFLQIPEDSITWVCDQIAESRGLDEESAIMWG
SFTSV CRGLLTEKRCMAAIREQVRPFLIFLQIPEDSISWVSDQFCDSRGLNEESTIMWG
HRTV CRLLLGRPTGRSNLREQVKPYLTLLQIREGDVNWVSEYKDDTRGLDEDSAEMWG

Figure S4 B

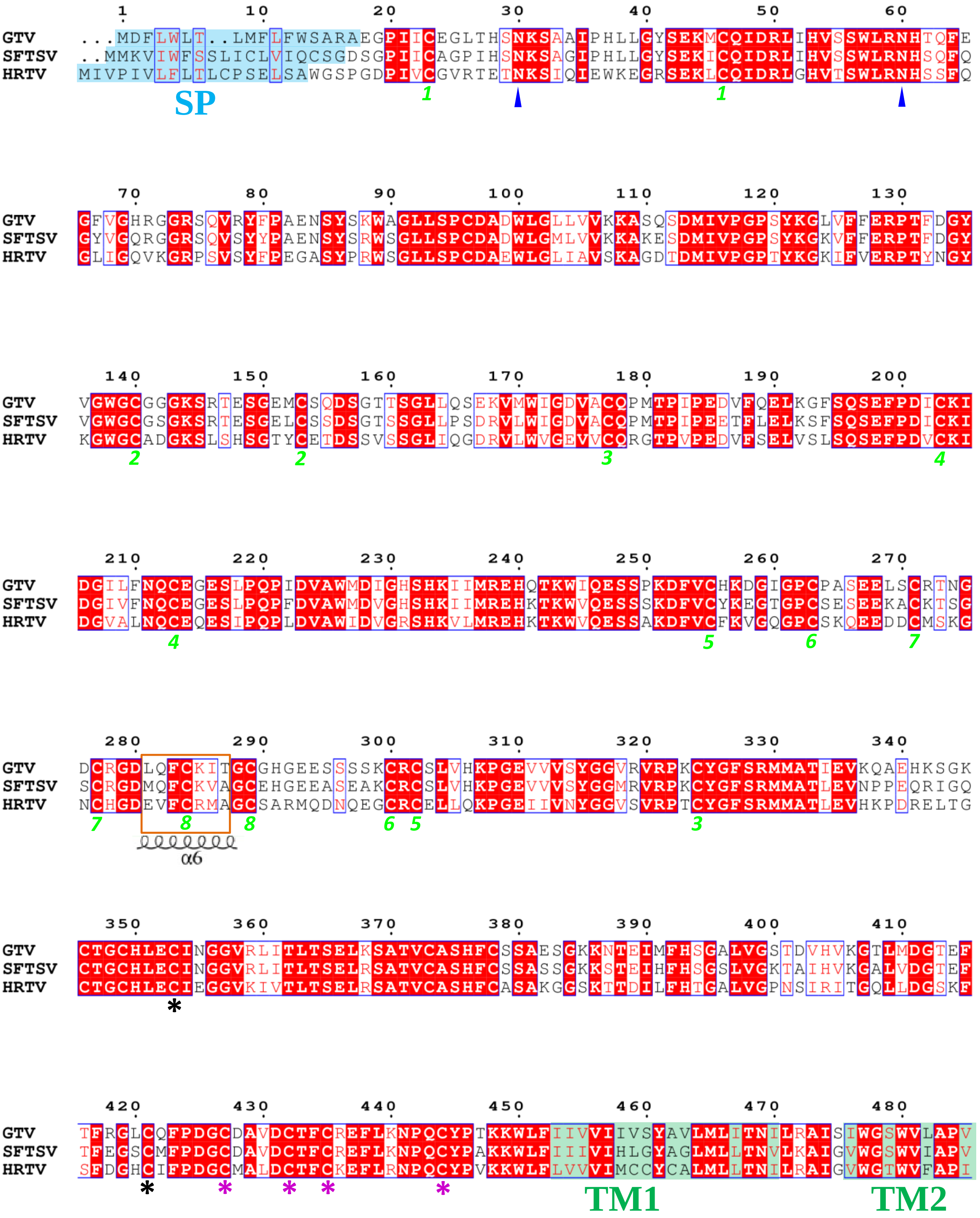

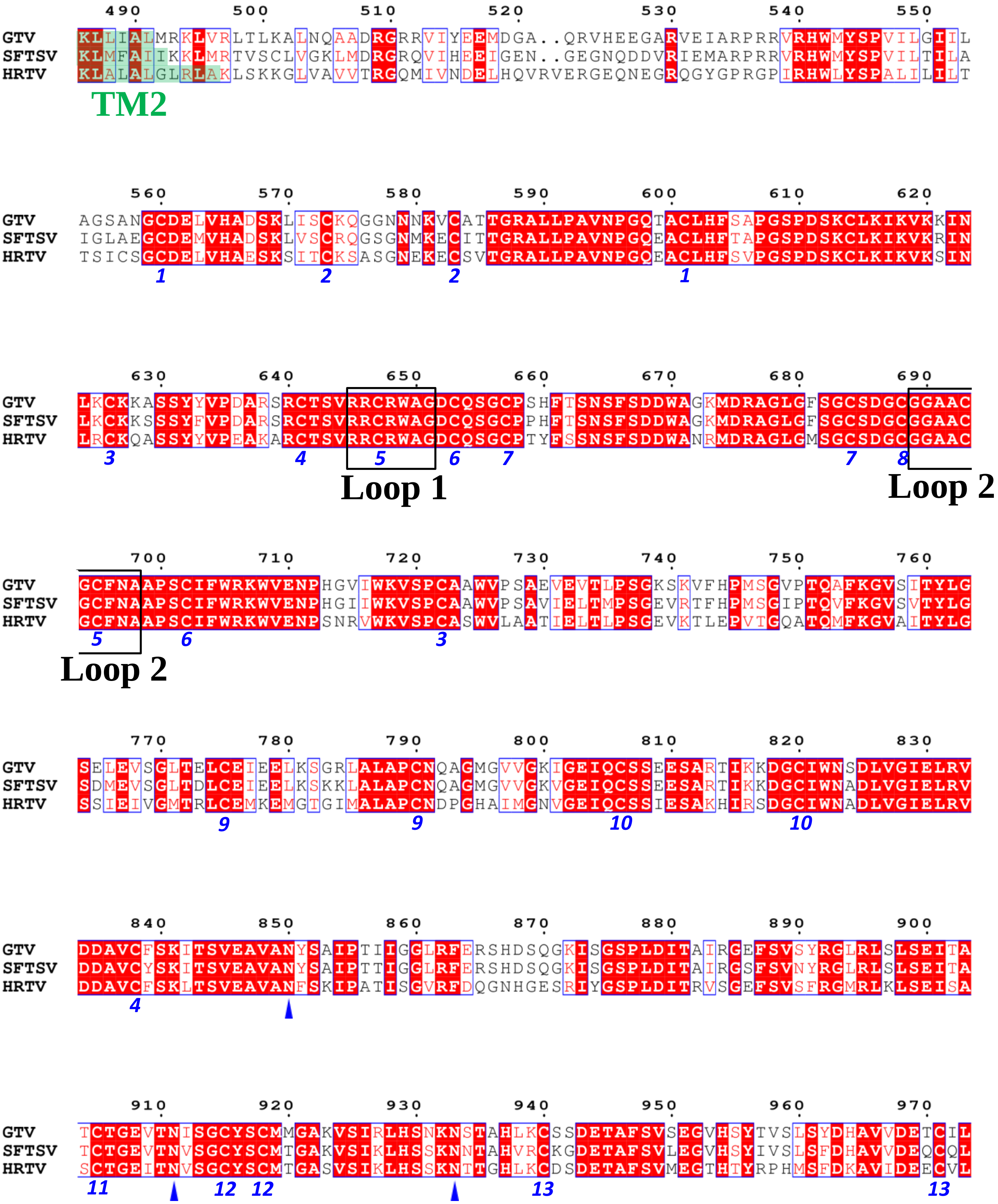
GTV
SFTSV
HRTV
TM2
Loop 1
Loop 2

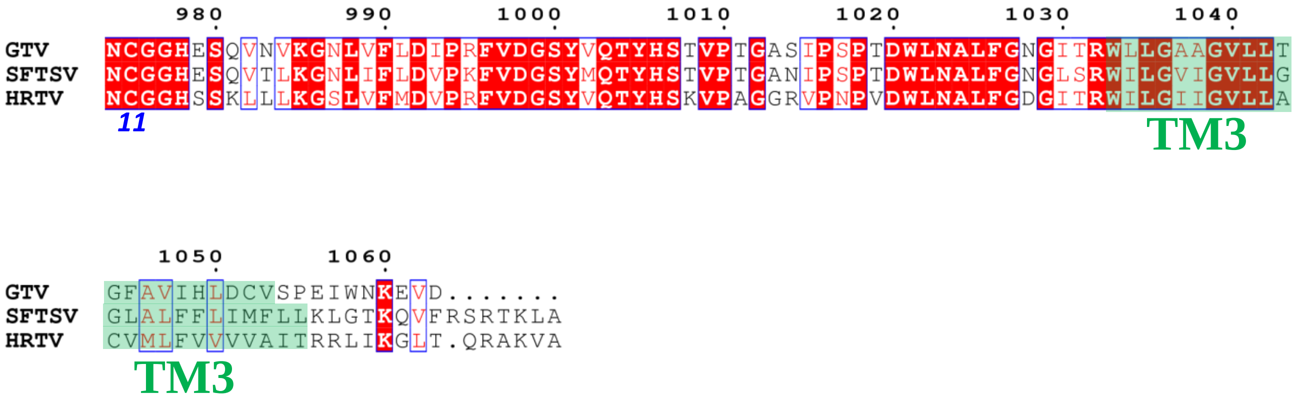
980 990 1000 1010 1020 1030 1040
GTV NCGGHESQVNVKGNLVFLDIPRFVDGSYVQTYHSTVPTGASIPSPTDWLNALFGNGITRWLLGAAGVLLT
SFTSV NCGGHESQVTLKGNLIFLDVPKFVDGSYMQTYHSTVPTGANIPSPTDWLNALFGNGLSRWILGVIGVLLG
HRTV NCGGHSSKLLLKGSLVFMDVPRFVDGSYVQTYHSKVPAGGRVPNPVDWLNALFGDGITRWILGIIGVLLA
11
TM3
1050 1060
GTV GFAVIHLDCVSPEIWNKEVD.......
SFTSV GLALFFLIMFLLKLGTKQVFRSRTKLA
HRTV CVMLFVVVVAITRRLIKGLT.QRAKVA
TM3

Figure S4 C

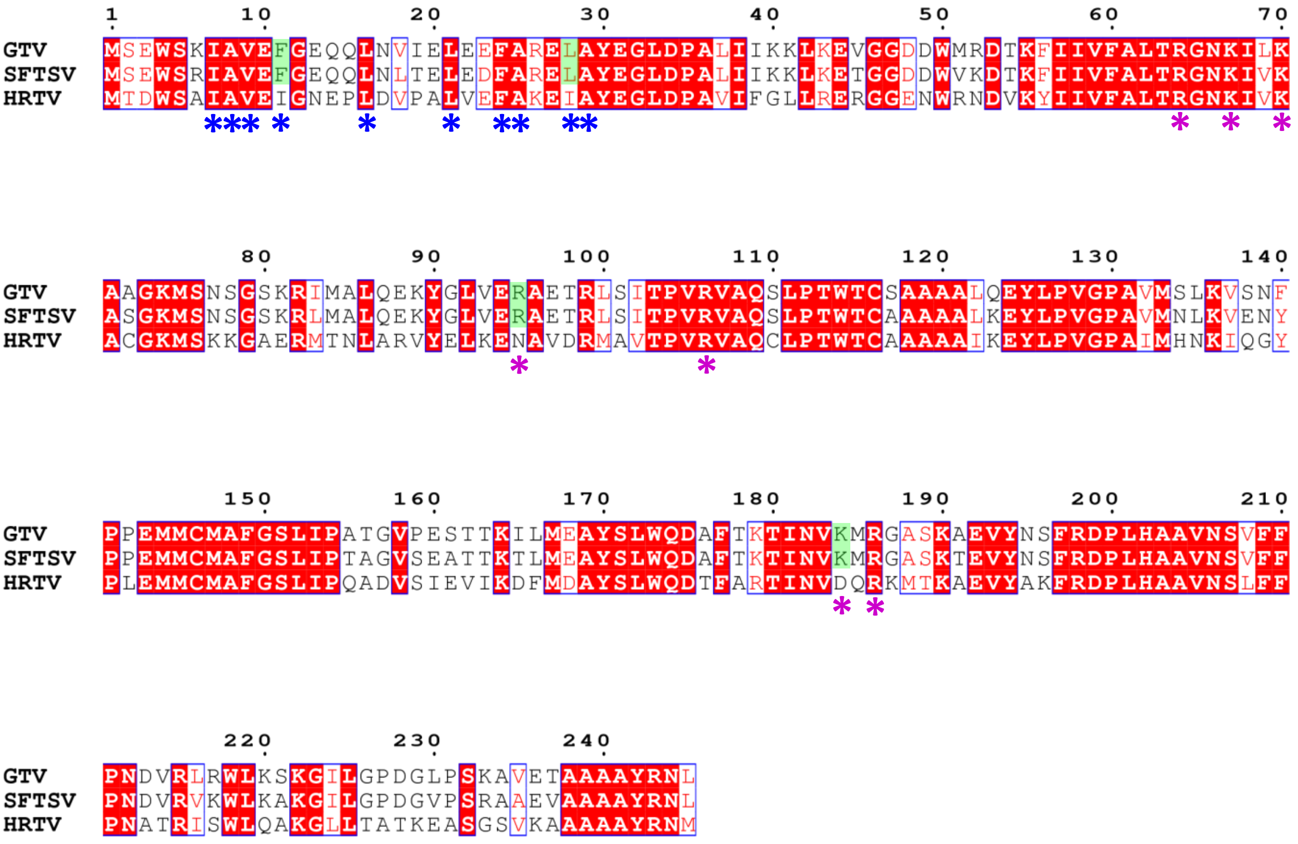
1 10 20 30 40 50 60 70
GTV MSEWSKIAVEFGEQQLNVIELEEFARELAYEGLDPALIIKKLKEVGGDDWMRDTKFIIVFALTRGNKILK
SFTSV MSEWSRIAVEFGEQQLNLTELEDFARELAYEGLDPALIIKKLKETGGDDWVKDTKFIIVFALTRGNKIVK
HRTV MTDWSAIAVEIGNEPLDVPALVEFAKEIAYEGLDPAVIFGLLRERGGENWRNDVKYIIVFALTRGNKIVK
80 90 100 110 120 130 140
GTV AAGKMSNSGSKRIMALQEKYGLVERAETRLSITPVRVAQSLPTWTCSAAAALQEYLPVGPAVMSLKVSNF
SFTSV ASGKMSNSGSKRLMALQEKYGLVERAETRLSITPVRVAQSLPTWTCAAAAALKEYLPVGPAVMNLKVENY
HRTV ACGKMSKKGAERMTNLARVYELKENAVDRMAVTPVRVAQCLPTWTCAAAAAIKEYLPVGPAIMHNKIQGY
150 160 170 180 190 200 210
GTV PPEMMCMAFGSLIPATGVPESTTKILMEAYSLWQDAFTKTINVKMRGASKAEVYNSFRDPLHAAVNSVFF
SFTSV PPEMMCMAFGSLIPTAGVSEATTKTLMEAYSLWQDAFTKTINVKMRGASKTEVYNSFRDPLHAAVNSVFF
HRTV PLEMMCMAFGSLIPQADVSIEVIKDFMDAYSLWQDTFARTINVDQRKMTKAEVYAKFRDPLHAAVNSLFF
220 230 240
GTV PNDVRLRWLKSKGILGPDGLPSKAVETAAAAYRNL
SFTSV PNDVRVKWLKAKGILGPDGVPSRAAEVAAAAYRNL
HRTV PNATRISWLQAKGLLTATKEASGSVKAAAAAYRNM

Figure S4 D

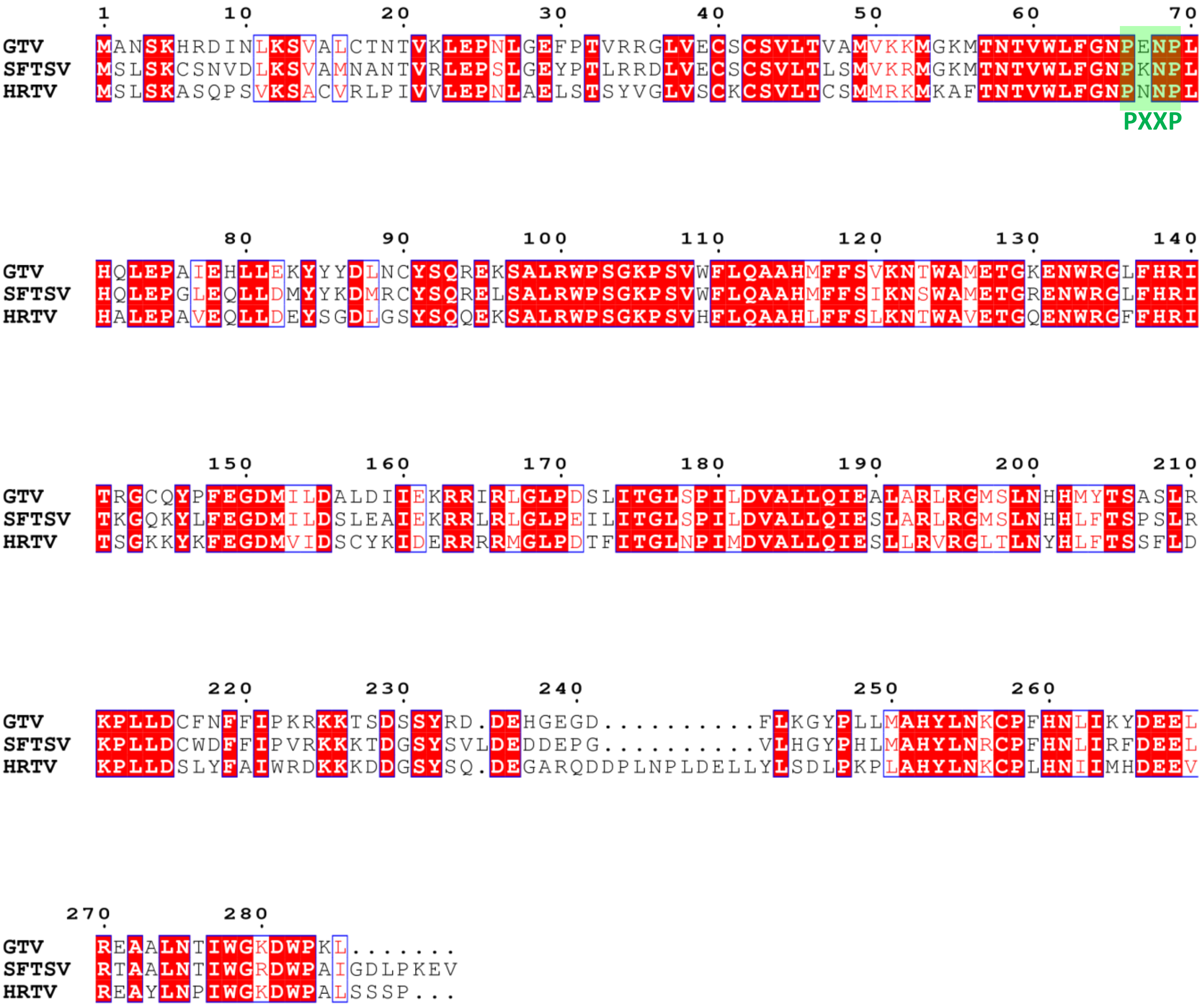
GTV MANSKHRDINLKSVALCTNTVKLEPNLGEFPTVRRGLVECSCSVLTVAMVKKMGKMTNTVWLFGNPENPL
SFTSV MSLSKCSNVDLKSVAMNANTVRLEPSLGEYPTLRRDLVECSCSVLTLSMVKRMGKMTNTVWLFGNPKNPL
HRTV MSLSKASQPSVKSACVRLPIVVLEPNLAELSTSYVGLVSCKCSVLTCSMMRKMKAFTNTVWLFGNPNNPL
PXXP
GTV HQLEPAIEHLLEKYYYDLNCYSQREKSALRWPSGKPSVWFLQAAHMFFSVKNTWAMETGKENWRGLFHRI
SFTSV HQLEPGLEQLLDMYYKDMRCYSQRELSALRWPSGKPSVWFLQAAHMFFSIKNSWAMETGRENWRGLFHRI
HRTV HALEPAVEQLLDEYSGDLGSYSQQEKSALRWPSGKPSVHFLQAAHLFFSLKNTWAVETGQENWRGFFHRI
GTV TRGCQYPFEGDMILDALDIIEKRRIRLGLPDSLITGLSPILDVALLQIEALARLRGMSLNHHMYTSASLR
SFTSV TKGQKYLFEGDMILDSLEAIEKRRLRLGLPEILITGLSPILDVALLQIESLARLRGMSLNHHLFTSPSLR
HRTV TSGKKYKFEGDMVIDSCYKIDERRRRMGLPDTFITGLNPIMDVALLQIESLLRVRGLTLNYHLFTSSFLD
GTV KPLLDCFNFFIPKRKKTSDSSYRD.DEHGEGD..........FLKGYPLLMAHYLNKCPFHNLIKYDEEL
SFTSV KPLLDCWDFFIPVRKKKTDGSYSVLDEDDEPG..........VLHGYPHLMAHYLNRCPFHNLIRFDEEL
HRTV KPLLDSLYFAIWRDKKKDDGSYSQ.DEGARQDDPLNPLDELLYLSDLPKPLAHYLNKCPLHNIIMHDEEV
GTV REAALNTIWGKDWPKL.......
SFTSV RTAALNTIWGRDWPAIGDLPKEV
HRTV REAYLNPIWGKDWPALSSSP...
